# Exploiting subtractive genomics to identify novel drug targets and new immunogenic candidates against *Bordetella pertussis*: an *in silico* study

**DOI:** 10.3389/fbinf.2025.1570054

**Published:** 2025-05-13

**Authors:** Mahshid Khazani Asforooshani, Narjes Noori Goodarzi, Behzad Shahbazi, Nayereh Rezaie Rahimi, Kimia Mahdavian, Mahdi Rohani, Farzad Badmasti

**Affiliations:** ^1^ Department of Bacteriology, Pasteur Institute of Iran, Tehran, Iran; ^2^ Department of Microbiology, Faculty of Biological Sciences, Alzahra University, Tehran, Iran; ^3^ Department of Pathobiology, School of Public Health, Tehran University of Medical Sciences, Tehran, Iran; ^4^ School of Pharmacy, Semnan University of Medical Sciences, Semnan, Iran; ^5^ Nervous System Stem Cells Research Center, Semnan University of Medical Sciences, Semnan, Iran; ^6^ Department of Environmental Health Engineering, School of Public Health, Shiraz University of Medical Sciences, Shiraz, Iran

**Keywords:** *Bordetella pertussis*, pertussis resurgence, immunogenic targets, multi-epitope vaccine, autotransporter proteins, reverse vaccinology

## Abstract

**Background:**

*Bordetella pertussis*, the causative agent of whooping cough, remains a significant global health concern despite the widespread availability of vaccines. The persistent reemergence of pertussis is driven by the bacterium’s ongoing genomic evolution, shifting epidemiological patterns, and limitations in current vaccine strategies. These challenges highlight the urgent need to identify novel drug targets and immunogenic candidates to enhance therapeutic and preventive measures against *B. pertussis*.

**Methods:**

Identification of novel drug targets and the detection of immunogenic factors as potential vaccine candidates were performed. Cytoplasmic proteins were evaluated for their similarity to the human proteome, metabolic pathways, and gut microbiota. On the other hand, surface-exposed proteins were evaluated as immunogenic targets using a reverse vaccinology approach. A multi-epitope vaccine (MEV) was designed based on the immunogenic linear B-cell epitopes of three autotransporters and the beta domain of SphB2 as a scaffold for MEV. Molecular docking, immune simulation results, and molecular dynamics simulations were performed to evaluate the binding affinity and feasibility of interaction between chimeric MEVs and immune receptors.

**Results:**

Six proteins were identified as excellent potential drug targets, including elongation factor P (WP_003810194.1), Aspartate kinase (WP_010930633.1), 50S ribosomal protein L21 (WP_003807462.1), Homoserine dehydrogenase (WP_003813074.1), Carboxynorspermidine decarboxylase (WP_003814461.1), and PTS sugar transporter subunit IIA (WP_010929966.1). On the other hand, reverse vaccinology identified nine immunogenic proteins, including BapA (WP_010930805.1), BrkA (WP_010931506.1), SphB2 (WP_041166323.1), TcfA (WP_010930243.1), FliK (WP_041166144.1), Fimbrial protein (WP_010930199.1), TolA (WP_010931418.1), DD-metalloendopeptidase (WP_003811022.1), and an I78 family peptidase inhibitor protein (WP_003812179.1). SphB2-based MEV was designed using six linear B-cell epitopes of the extracellular loops of the autotransporters. The binding affinity and feasibility of the interaction between MEV and TLR2, TLR4, and HLA-DR-B were computationally confirmed by molecular dynamics.

**Conclusion:**

It appears that proteins involved in translation and metabolism can be considered novel drug targets. Furthermore, this study highlights autotransporter proteins as promising immune targets. There is no doubt that experimental work should be conducted to confirm the results in the future.

## 1 Introduction

*Bordetella pertussis* is a Gram-negative bacterium that causes whooping cough in humans. The incidence rates of pertussis disease among infants younger than 2–3 months old are significant across all countries with available data, exceeding 1,000 cases per 100,000 population during outbreaks (Kandeil et al., 2020). Moreover, its incidence in adults aged older than 50 years is significantly higher than that of the general population (See, 2025). Considering its high frequency, pertussis requires continuous and high coverage of vaccination in all age groups to control its associated mortality and morbidity.

Despite continuous vaccination with whole-cell pertussis (DTwP) or acellular pertussis (DTaP) vaccines worldwide, there has been a surprising resurgence of this disease. Low vaccination coverage, low anti-pertussis herd immunity in target population, antigenic drift, genetic shift, and gene loss in pertussis pathogens (e.g., novel PT promoter allele of pertussis toxin, and pertactin-deficient isolates), epidemiological changes (circulating of new clones) under vaccine selection pressure, and lower efficacy of the DTaP compared with the DTwP are the main causes of pertussis resurgence (Nian et al., 2023; Fullen et al., 2020). Traditional methods appear inadequate for controlling this highly infectious disease, highlighting the need for state-of-the-art innovations.

The continued advancement of pertussis vaccine technology remains of significant importance owing to the drawbacks associated with both DTwP and DTaP, such as unwanted immunogenic reactions due to the presence of high levels of LPS in DTwP and non-stimulation of the Th1 cellular immune system by DTaP formulation (Guiso et al., 2020; Saso et al., 2021). Thus, in recent years, to address the inadequacy of the aforementioned vaccines in stimulating mucosal responses and IgA secretion, a new genetically modified attenuated vaccine called Vaccine-BPZE1 has been developed for administration via inhalation (Thorstensson et al., 2014; Brennan, 2017). However, concerns regarding its safety in infants remain a notable weakness.

Many efforts are underway to identify the best immunogenic targets, adjuvants, and new routes of immunization for protection against pertussis. Novel experimental vaccine formulations, such as outer membrane vesicles (OMV), aP mixed with different adjuvants (e.g., cholera toxin, curdlan, TLR9 agonist CpG, STING agonist c-di-GMP, monophosphoryl lipid A and lipooligosaccharides), and other virulence factors, including adenylate cyclase toxin (ACT), BrkA, SphB1, SphB2, BatB, and Vag8, have been proposed (Chasaide and Mills, 2020; Locht, 2021). On the other hand, due to the increased prevalence of high-level macrolide resistant strains, there is a need for drug development against pertussis (Ivaska et al., 2022a; Feng et al., 2021; Kaur et al., 2019).

Several experimental and computational analyses have been performed to inhibit pertussis toxin and to introduce novel drug targets, respectively (Ernst, 2022; Jamal et al., 2022). Immunoinformatics and reverse vaccinology (RV) help identify new potentially immunogenic proteins. In this regard, RV enables researchers to use whole bacterial proteins to identify optimal candidates and characterize them in terms of antigenicity, allergenicity, and other relevant aspects (Noori Goodarzi et al., 2023). This may lead to the development of a new generation of vaccines based on novel or even overlooked antigens. It seems that the genomics-based approach has demonstrated its ability to identify potential vaccine and drug candidates. For example, RV effectively contributed to the advancement of the 4CMenB vaccine (Bexsero, GSK). This vaccine is the first approved immunization against *Neisseria meningitidis* serogroup B (MenB), currently authorized in European countries (Rappuoli et al., 2018).

Multi-epitope vaccines (MEVs) represent a promising advancement in immunization strategies, leveraging the ability to elicit robust immune responses against various pathogens through the incorporation of multiple epitopes within a single vaccine construct. This approach is particularly advantageous over traditional monovalent vaccines, as it can activate both humoral and cellular immune responses, thereby enhancing overall immunogenicity and efficacy (Al-Zayadi et al., 2024). MEVs have shown immunoreactivity against bacterial pathogens including *Brucella melitensis* (Li et al., 2022), and uropathogenic *Escherichia coli* (Rezaei et al., 2023).

The aim of this study was to introduce novel targets with suitable characteristics as new drug targets and potential vaccine candidates to facilitate the advancement of new preventive and therapeutic approaches against pertussis. The identification of immunogenic epitopes will be used to develop a multi-epitope chimeric vaccine candidate. This study provides a basis for inventing a new generation of vaccines using an interdisciplinary field.

## 2 Methods

### 2.1 Identification of novel drug targets

#### 2.1.1 Genome retrieval and core proteome determination

In this study, 554 completed genomes of *B. pertussis* were selected from the EDGAR 3.0 database (https://edgar3.computational.bio.uni-giessen.de/cgi-bin/edgar_login.cgi) as a repository of bacterial genomes. The name of selected strains is listed in the Supplementary Table S1. The database was subjected to identifying core proteins available in all genomes. Core proteins were extracted in FASTA file for further analysis.

#### 2.1.2 Identification of cytoplasmic proteins using PSORTb

The subcellular localization of the core proteins was performed using PSORTb (https://www.psort.org/psortb/). This software is one of the most precise bacterial localization prediction tools (Yu et al., 2010). Based on the predictions for the proteins, all cytoplasmic proteins were collected. It appears that cytoplasmic proteins are suitable drug targets because of their vital roles in growth and metabolic pathways (Khan et al., 2022).

#### 2.1.3 Sequence similarity of proteins against human proteomes

The cytoplasmic proteins were checked for sequence similarity against the human proteome (taxid: 9,606) using PSI-BLAST (https://blast.ncbi.nlm.nih.gov/Blast.cgi) (Mahram and Herbordt, 2015). According to the default criteria, all proteins exhibiting any similarity to human proteins were excluded from the study.

#### 2.1.4 Identification of pertussis-specific metabolomic proteins

Proteins involved in common metabolic roles between bacteria and humans were removed from the analysis using the KEGG Automatic Annotation Server (https://www.genome.jp/kegg/kaas/). On the other hand, Pertussis-specific proteins were extracted from the FASTA file for further evaluation. Proteins retained from the previous step were subjected to functional analysis via the KAAS server, allowing for a comparative assessment between the *B. pertussis* and human proteomes to identify non-homologous, pathogen-specific targets.

#### 2.1.5 Removal of human mitochondrial homolog proteins

Human mitochondrial homolog proteins were detected using the MITOMASTER database (https://www.mitomap.org/foswiki/bin/view/MITOMASTER/WebHome) based on default BLAST parameters. Non-homologous proteins were considered for further investigation.

#### 2.1.6 Identification of essential proteins

Essential proteins were detected using Genome BLAST against the DEG database (http://origin.tubic.org/deg/public/index.php/index). This database has collected all essential proteins involved in the vital roles of bacterial life based on experimental studies (Luo et al., 2021). Proteins with an identity ≥60 and coverage ≥80% were selected as essential proteins.

#### 2.1.7 Identification of novel targets using DrugBank

In this study, we assessed the sequence similarity of the drug targets to those in the DrugBank database (https://go.drugbank.com/) based on default BLAST parameters. We assumed that any protein with no sequence similarity with the characterized drug targets in this database was considered a novel target.

#### 2.1.8 Protein data bank availability

The proteins were screened for PDB file availability using the Protein Data Bank (https://www.rcsb.org/). Proteins with at least 80% coverage and 50% identity with previously characterized PDB files were identified as feasible drug targets for further analysis. This threshold was used as an acceptable criterion for selecting proteins suitable for homology modeling and downstream drug target evaluation.

#### 2.1.9 Evaluation of sequence similarity with gut microbiota

The proteins were analyzed for sequence similarity with the gut microbiota (including 83 gut microbiome strains) using the NCBI BLAST search, and proteins with an identity ≥50% and coverage ≥70% were excluded from the study. The gut microbiota strains were selected based on previous studies (Chakkyarath et al., 2021; Shanmugham and Pan, 2013). See the list of the gut microbiome in the Supplementary Table S2.

#### 2.1.10 RNA-seq data analysis

The paired-end fastq files of *B. pertussis* strain CS were downloaded from the EBI database (https://www.ebi.ac.uk/ena/browser/view/SRR27664439). This RNA-seq was obtained from the lungs of mice infected with the CS strain. The data were analyzed by CLC Genomics version 20 (with defult parameters) and the expressions were reported as the transcript per million (TPM).

### 2.2 Identification of immunogenic targets as potential vaccine candidates

#### 2.2.1 Protein collection

The core proteomes of 554 *B. pertussis* strains were considered for subcellular localization analysis. Additionally, 11 confirmed virulence factors plus 3,664 proteins from the *B. pertussis* strain CS (accession number: CP002695) were incorporated into this analysis (Zhang et al., 2011). Next, redundant proteins were excluded (identity ≥90%) using Jalview software, and the remaining proteins were collected for further analysis.

#### 2.2.2 Prediction of protein localization in bacterial cells

All non-redundant sequences were submitted to the PSORTb database to predict the subcellular localization of core proteins. This step identified extracellular, outer membrane, and surface-exposed proteins. Subsequently, TMHMM Server v. 2.0 (https://services.healthtech.dtu.dk/services/TMHMM-2.0/2-Guide.php) was employed to exclude transmembrane proteins that remained (Käll et al., 2007).

#### 2.2.3 Consecutive and comparative analysis

The immunogenic potential of the candidate proteins was assessed using the VaxiJen tool (http://www.ddgpharmfac.net/vaxijen/VaxiJen/VaxiJen.html)) (Doytchinova and Flower, 2007). A cutoff value of ≥0.5 was employed to identify potentially antigenic proteins. Conversely, the allergenicity of these proteins was evaluated using AlgPred 2.0 (http://crdd.osdd.net/raghava/algpred/), with a threshold value of ≥0.3 (Sharma et al., 2021). Following the prediction steps, the sequence similarity of all selected proteins to host proteins (*Homo sapiens*, TaxID: 9,606) was analyzed using the PSI-BLAST tool in the BLASTp database. Proteins exhibiting similarity to the host proteome were excluded from further analyses.

Next, protein characterization and domain analysis were performed. To determine the functional class of the selected proteins, the VICMpred database (https://webs.iiitd.edu.in/raghava/vicmpred/submission.html) was employed (Saha and Raghava, 2006). Subsequently, the Expasy ProtParam server (https://www.expasy.org/protparam) was used to evaluate the physicochemical properties of the proteins (Roy et al., 2011). Additionally, protein domain identification was performed using two resources: the Conserved Domain Database (CDD) (https://www.ncbi.nlm.nih.gov/Structure/cdd/cdd.shtml) and EggNOG (http://eggnog5.embl.de/). CDD, part of the NCBI’s Entrez system, provides protein sequence annotation with conserved domain positions (Marchler-Bauer et al., 2005). EggNOG offers an annotated orthology resource encompassing over 5,090 organisms and 2,502 viruses (Huerta-Cepas et al., 2016). Finally, the prevalence of each protein was determined among the 554 completed genomes. Proteins with a prevalence ≥75% were considered in this step.

#### 2.2.4 Immunoinformatic analyses

##### 2.2.4.1 Detection of linear B-cell epitopes and human MHC II binding sites

Linear B-cell epitopes of the selected proteins were evaluated using the BepiPred-2.0 tool (https://services.healthtech.dtu.dk/services/BepiPred-2.0/4-Abstract.php). A threshold of ≥0.6 was employed for epitope identification (Jespersen et al., 2017). The B cell epitope ratio, defined as the number of amino acids within an epitope divided by the total protein length, was calculated for each protein. Proteins exceeding the average ratio were selected. In the following step, TepiTool, a resource available in the IEDB (http://tools.iedb.org/tepitool/), was employed to predict human MHC-II binding sites. A cutoff corresponding to the top 5% of the predicted peptides was applied (Paul et al., 2016). The MHC-II binding site ratio was calculated as the number of predicted binding sites divided by the total protein length and determined for each protein.

The selected proteins were then analyzed using a quartile scoring method that incorporated three distinct indicators: antigenicity, linear B cell epitope ratio, and MHC-II binding site ratio. The final protein scores were determined by summing the individual scores across all the indicators.

##### 2.2.4.2 Homology modeling and characterization of conformational epitopes

The tertiary structure (3D) of the immunogenic proteins was predicted using homology modeling. The AlphaFold Protein Structure Database (https://alphafold.ebi.ac.uk) (Jumper et al., 2021) was selected for this purpose. The accuracy of the 3D model was validated by an assessment on the ProSA web server (https://prosa.services.came.sbg.ac.at/prosa.ph) (Wiederstein and Sippl, 2007), which identified potential errors in the 3D model. Ramachandran plots on the Zlab Ramachandran Plot Server (https://zlab.umassmed.edu/bu/rama/index.pl) (Anderson et al., 2005) were used to predict energetically rejected and allowed regions. ElliPro (http://tools.iedb.org/ellipro/) was used to detect all conformational B-cell epitopes using a threshold of ≥0.8 (Ponomarenko et al., 2008). PyMOL software version 2.3.4 was used to visually display the conformational B-cell epitopes on the surface of each protein in different colors (Rosignoli and Paiardini, 2022).

#### 2.2.5 Selection of shortlisted proteins

Final promising immunogenic targets were identified using the quartile scoring method (Noori Goodarzi et al., 2021). Proteins with a score ≥8 were selected as shortlisted proteins in this study.

#### 2.2.6 Designing chimeric multi-epitope vaccines

##### 2.2.6.1 Best linear B-cell epitopes

Linear B-cell epitopes anticipated to reside on the extracellular loops of the three selected outer membrane proteins were identified using the BepiPred tool, with a cut-off value of ≥0.6. Furthermore, the IEDB Analysis Resource web tool (http://tools.iedb.org/conservancy/) was used to assess the level of conservation within the detected linear B-cell epitopes.

##### 2.2.6.2 Implantation of selected linear B-cell epitopes on a platform

Epitopes from the three selected proteins were evaluated based on antigenicity, allergenicity, human protein homology, conservation, and surface accessibility to identify the most immunogenic candidates for an effective multi-epitope vaccine (MEV). The β-barrel domain of SphB2 protein (WP_240079968.1) was chosen as a scaffold to optimizing its presentation to the immune system. The tertiary structure of the chimeric protein was modeled using the SWISS-MODEL web tool (https://swissmodel.expasy.org/). The reliability of this model was validated using the ProSA online tool and Ramachandran plot analysis.

##### 2.2.6.3 Immune simulation and molecular docking of MEV

To evaluate the binding affinities of MEV toward human TLR2 (PDB: 2Z7X_A), TLR4 (PDB: 3FXI_A), and HLA-DR-B (PDB: 3PDO_B), molecular docking was conducted using pyDockWEB (https://life.bsc.es/pid/pydockweb/default/index). Additionally, an immunoreactivity simulation (a single dose of injection) of multi-epitope vaccines was performed using C-ImmSim (https://kraken.iac.rm.cnr.it/C-IMMSIM/index.php) (Fereshteh et al., 2023). Finally, the PDBsum online tool (http://www.ebi.ac.uk/thornton-srv/databases/pdbsum/) was employed to identify the residues paired with TLR2, TLR4, and HLA-DR-B (Laskowski, 2001).

##### 2.2.6.4 Molecular dynamics simulations

Molecular dynamics (MD) simulations were conducted to determine the feasibility of the interactions between chimeric MEV and TLR2, TLR4, and HLA-DR-B complexes using GROMACS version 2019 software (Abraham et al., 2015). The simulations utilized the Optimized Potential for Liquid Simulations (OPLS) force field to investigate the stability and conformational changes of MEV in both its free state and when complexed with the receptors. Each complex was positioned within a 10 Å solvent box containing simple point charge (SPC) water molecules. To achieve system neutrality, a suitable number of Na^+^ and Cl^−^ ions were added. The energy minimization for all MD simulations consisted of two phases: the systems underwent equilibration for 100 picoseconds (ps) under NVT (constant number of particles, volume, and temperature) and NPT (constant number of particles, pressure, and temperature) conditions. The Parrinello–Rahman barostat was employed to maintain a steady temperature of 300 K and pressure of 1.0 bar. The particle mesh Ewald (PME) method managed long-range electrostatics with a 10 Å cutoff distance and grid spacing of 0.16 nm. A 1 nm cutoff was used for van der Waals (VDW) interactions. The LINCS algorithm was implemented to constrain the lengths of covalent bonds. Each simulation was performed for a total duration of 100 nanoseconds (ns), with a time step of two femtoseconds (fs). Post-simulation analysis of system stability was carried out using root mean square deviation (RMSD) and radius of gyration (Rg). The root-mean-square fluctuation (RMSF) was used to assess the fluctuation of individual amino acids throughout the simulation, and the number of hydrogen bonds in each protein-protein complex was also examined (Shahbazi et al., 2023).

## 3 Results

### 3.1 *In silico* identification of potential drug targets

**FIGURE 1:**
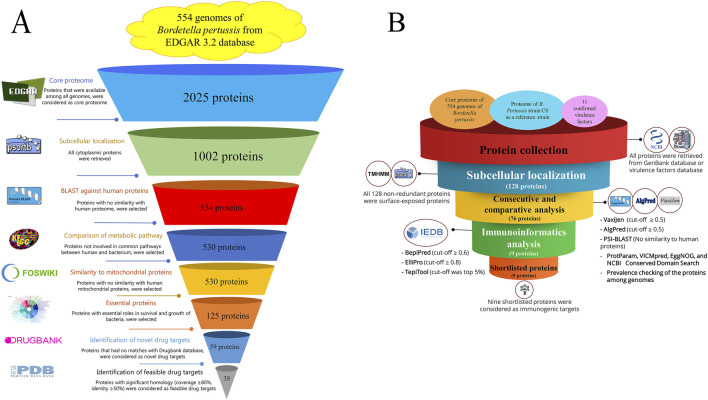
**(A)** Step-by-step flowchart for identifying novel drug targets among the 554 *B. pertussis* genomes. The core proteome was obtained using EDGAR 3.2, and cytoplasmic proteins were detected using PSORTb v3.0. Protein sequences that showed a similarity with the human proteome (taxid: 9,606) were excluded using Position-Specific Iterated protein BLAST. KAAS (KEGG Automatic Annotation Server) was applied to remove proteins involved in common metabolic pathways between humans and bacteria. The MITOMASTER tool was used to detect proteins similar to human mitochondrial proteins. Essential proteins were selected using the DEG database, and the druggability of the proteins was checked using the DRUGBANK database. Novel drug targets were evaluated to identify the same or relevant pdf file in the Protein Data Bank server. **(B)** Flowchart of the reverse vaccinology process in *B. pertussis*. A collection of proteins, including a core proteome (extracted from 554 genome using EDGAR), the proteome of *the B. pertussis* CS strain (GenBank accession number: CP086368), and 11 confirmed virulence factors collected from Virulence Factor Database (http://www.mgc.ac.cn/cgi-bin/VFs/genus.cgi?Genus=Bordetella), were obtained. Non-redundant proteins were subjected to the PSORTb tool to determine the subcellular localization of the proteins. Surface-exposed and secretory proteins were evaluated using several consecutive analyses. Total antigenicity (VaxiJen v2.0), allergenicity (AlgPred 2.0), similarity to human proteins (protein BLAST), role prediction (VICMpred), domain characterization (EggNOG and CD domain search), and prevalence evaluation (using local BLAST) were performed. Proteins with all acceptable criteria were subjected to immunoinformatics analysis. Linear and conformational epitopes (using PepiPred and ElliPro) along with MHC II binding sites (TepiTool). Shortlisted proteins were considered promising immunogenic targets.

**FIGURE 2:**
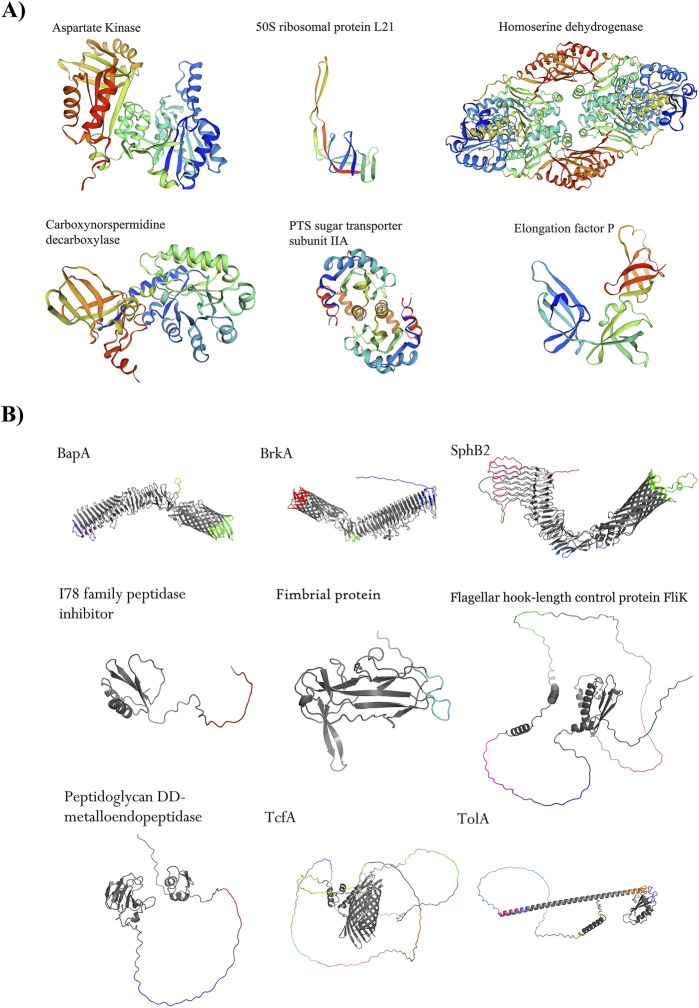
Shortlisted proteins as novel drug targets and immunogenic proteins. **(A)** Six proteins, such as Aspartate kinase (WP_010930633.1), 50S ribosomal protein L21 (WP_003807462.1), homomerine dehydrogenase (WP_003813074.1), PTS sugar transporter subunit IIA (WP_010929966.1), Carboxynorspermidine decarboxylase (WP_003814461.1), and elongation factor P (WP_003810194.1) are potential excellent drug targets. **(B)** Nine shortlisted immunogenic proteins are promising. Four autotransporter proteins, namely BapA (GenBank accession number is WP_010930805.1), BrkA (WP_010931506.1), SphB2 (WP_041166323.1), and TcfA (WP_010930243.1), were detected as the top immunogenic targets. Adhesion proteins such as hook-filament junction FliK (WP_041166144.1), fimbrial protein (WP_010930199.1), and TolA (WP_010931418.1) were among the shortlisted proteins. A lipoprotein with enzymatic activity including peptididoglycan DD-metalloendopeptidase (WP_003811022.1) and an I78 family peptidase inhibitor protein (WP_003812179.1) showed promising criteria as immunogenic targets. All conformational epitopes (ElliPro tool, threshold ≥0.8) were depicted in different colors on protein structures.

The step-by-step process of novel drug target identification is shown in Figure 1A. In this study, 2025 proteins were detected as core proteomes among 554 completed genomes of *B. pertussis* strains. In total, 1,002 proteins were identified as cytoplasmic proteins based on subcellular localization analysis. Among these proteins, 534 were not similar to human proteins. Next, 530 were unique to the *B. pertussis* metabolic pathways. Based on the DEG results, 125 proteins were considered essential for the survival and growth of the bacterium. The DrugBank results identified 59 novel drug targets (see the Supplementary Table S3). Based on the PDB availability results, 38 feasible drug targets were selected. See Table 1. In addition, the FASTA file of the 38 feasible drug targets was deposited in the Supplementary Material S4. Moreover, the expression profile of all genes was measured and reported based on the transcript per million (TPM) (See the Supplementary Material S5). Finally, six proteins were considered the best targets after assessing the sequence similarity of the proteins with the proteomes of the gut microbiota. These six proteins, including elongation factor P (WP_003810194.1), Aspartate kinase (WP_010930633.1), 50S ribosomal protein L21 (WP_003807462.1), Homoserine dehydrogenase (WP_003813074.1), Carboxynorspermidine decarboxylase (WP_003814461.1), and PTS sugar transporter subunit IIA (WP_010929966.1), were promising drug targets. Figure 2A shows the results.

**TABLE 1:** The shortlisted novel drug targets from *B. pertussis*.

No.	Protein	Accession number	Protein target	PDB target	Coverage	Identity	Gut microbiota coverage	Gut microbiota Identity	Gut microbiota Blast	Expression*
1	UMPkinase	WP_010930349.1	Chain A, Uridylate kinase [*Neisseria meningitidis*]	1YBD_A	97%	66.52%	97	56	+	337.83
2	Imidazole glycerol phosphate synthase subunit HisF	WP_003815802.1	Chain A, HisF-LUCA [synthetic construct]	4EVZ_A	94%	67,84%	94	64	+	224.28
3	50S ribosomal protein L35	WP_003812834.1	Chain 2, 50S ribosomal protein L35 [*Acinetobacter baumannii* AB0057]	6V39_2	98%	59.38%	98	64	+	2905.52
4	Elongation factor P	WP_003810194.1	Chain A, Elongation factor P [*Pseudomonas aeruginosa*]	3OYY_A	99%	63.44%	100	38	-	2170.51
5	50S ribosomal protein L35	WP_003812834.1	Chain 2, 50S ribosomal protein L35 [*Acinetobacter baumannii* AB0057]	6V39_2	98%	59.38%	98	64	+	2905.52
6	50S ribosomal protein L19	WP_003813315.1	Chain A, Translation initiation factor IF-3 [*Escherichia coli*]	8JSG_A	94%	69.94%	93	64	+	2105.99
7	Imidazoleglycerol-phosphate dehydratase HisB	WP_003815795.1	Chain A, Imidazoleglycerol-phosphate dehydratase 1 [*Arabidopsis thaliana*]	2F1D_A	99%	52.58%	99	56	+	617.93
8	50S ribosomal protein L29	WP_003806912.1	Chain A, 50S ribosomal protein L29 [*Pseudomonas aeruginosa* PAO1]	7UNR_1	100%	55.56%	98	59	+	709.65
9	50S ribosomal protein L18	WP_003806922.1	Chain Nc, Large ribosomal subunit protein uL18 [*Psychrobacter urativorans*]	8RD8_Nc	100%	60.83%	100	56	+	917.53
10	30S ribosomal protein S21	WP_006218592.1	Chain v, 30S ribosomal protein S21 [*Escherichia coli* K-12]	7QG8_v	92%	63.08%	100	60	+	5543.98
11	Translation initiation factor IF-3	WP_033446215.1	Chain O, 50S ribosomal protein L19 [*Acinetobacter baumannii* AB0057]	6V39_O	81%	74.76%	94	69	+	1025.92
12	30S ribosomal protein S6	WP_010926331.1	Chain f, 30S ribosomal protein S6 [*Acinetobacter baumannii* AB0057]	6V39_f	94%	60.50%	99	59	+	2185.46
13	Protein phosphatase CheZ	WP_003817162.1	Chain Z, Chemotaxis protein cheZ [*Escherichia coli*]	1KMI_Z	93%	65.50%	94	65	+	0
14	50S ribosomal protein L24	WP_003806917.1	Chain U, 50S ribosomal protein L24 [*Escherichia coli* K-12]	3J7Z_U	93%	58.59%	93	58	+	2452.47
15	50S ribosomal protein L33	WP_010930711.1	Chain 1, 50S ribosomal protein L33 [*Escherichia coli* K-12]	3J7Z_1	100%	76.36%	100	78	+	2973.79
16	Ketol-acid reductoisomerase	WP_010930015.1	Chain A, Ketol-acid reductoisomerase [*Pseudomonas aeruginosa*]	1NP3_A	100%	71.01%	98	59	+	178.63
17	Integration host factor subunit alpha	WP_010931006.1	Chain A, protein (integration host factor (alpha) (IHF)) [*Escherichia coli*]	1IHF_A	88%	68.04%	83	69	+	309.86
18	Aspartate kinase	WP_010930633.1	Chain A, Aspartokinase [*Pseudomonas aeruginosa* PAO1]	5YEI_A	98%	63.13%	98	46	-	837.08
19	Transcription termination/antitermination protein NusG	WP_003806885.1	Chain A, Transcription antitermination protein NusG [*Escherichia coli*]	2JVV_A	98%	60.00%	98	60	+	992.27
20	ATP-dependent protease subunit HslV	WP_010931291.1	Chain A, ATP-dependent protease subunit HslV [*Escherichia coli* 55989]	5JI2_A	95%	67.25%	96	65	+	280.35
21	50S ribosomal protein L36	WP_003806928.1	Chain 6, 50S ribosomal protein L36 [*Pseudomonas aeruginosa*]	6SPB_6	100%	73.68%	100	78	+	796.80
22	Transcriptional repressor LexA	WP_010930566.1	Chain A, LexA repressor [*Escherichia coli* K-12]	7B5G_A	96%	58.85%	96	58	+	348.83
23	50S ribosomal protein L6	WP_010931568.1	Chain G, 50S ribosomal protein L6 [*Pseudomonas aeruginosa* PAO1]	7UNR_G	100%	59.32%	100	59	+	1445.89
24	50S ribosomal protein L21	WP_003807462.1	Chain Q, 50S ribosomal protein L21 [*Acinetobacter baumannii* AB0057]	6V39_Q	100%	59.22%	100	47	-	5677.25
25	50S ribosomal protein L23	WP_003806906.1	Chain V, 50S ribosomal protein L23 [*Pseudomonas aeruginosa* PAO1]	7UNR_V	96%	52.63%	92	54	+	1478.25
26	GTP cyclohydrolase FolE2	WP_003813103.1	Chain A, GTP cyclohydrolase FolE2 [*Burkholderia pseudomallei*]	8G6C_A	98%	61.15%	95	50	+	493.26
27	Phosphoribosyl-AMP cyclohydrolase	WP_010931645.1	Chain A, Phosphoribosyl-AMP cyclohydrolase [*Methanothermobacter thermautotrophicus*]	1ZPS_A	86%	55.17%	94	52	+	261.66
28	Homoserine dehydrogenase	WP_003813074.1	Chain A, Homoserine dehydrogenase [*Thiobacillus denitrificans* ATCC 25259]	3MTJ_A	94%	69.90%	100	45	-	185.61
29	Translation initiation factor IF-1	WP_003806927.1	Chain A, Translation initiation factor IF-1 [*Burkholderia thailandensis* E264]	2N3S_A	100%	94.44%	94	73	+	483.90
30	Acetolactate synthase small subunit	WP_003814006.1	Chain A, Probable acetolactate synthase isozyme III (Small subunit) [*Nitrosomonas europaea* ATCC 19718]	2PC6_A	99%	66.67%	99	58	+	338.48
31	50S ribosomal protein L28	WP_003810297.1	Chain Z, 50S ribosomal protein L28 [*Pseudomonas aeruginosa* PAO1]	7UNR_Z	97%	78.95%	99	76	+	1341.46
32	Chemotaxis protein CheW	WP_003811919.1	Chain A, Chemotaxis protein CheW [*Escherichia coli*]	2HO9_A	100%	70.48%	100	72	+	0
33	Preprotein translocase subunit SecA	WP_003814564.1	Chain A, Preprotein translocase SecA subunit [*Escherichia coli*]	2FSF_A	92%	60.17%	99	58	+	702.73
34	Carboxynorspermidine decarboxylase	WP_003814461.1	Chain A, Putative carboxynorspermidine decarboxylase protein [*Sinorhizobium meliloti*]	3MT1_A	99%	67.03%	100	42	-	124.09
35	Flavodoxin-dependent (E)-4-hydroxy-3-methylbut-2-enyl-diphosphate synthase	WP_010930793.1	Chain A, 4-hydroxy-3-methylbut-2-en-1-yl diphosphate synthase [*Thermus thermophilus* HB8]	4S38_A	93%	60.20%	94	39	-	198.58
36	PTS sugar transporter subunit IIA	WP_010929966.1	Chain A, PTS IIA-like nitrogen-regulatory protein PtsN [*Burkholderia pseudomallei* 1710b]	4GQX_A	99%	65.33%	85	38	-	298.80
37	50S ribosomal protein L32	WP_003813827.1	Chain 4, 50S ribosomal protein L32 [*Pseudomonas aeruginosa* PAO1]	7UNR_4	100%	65.00%	90	61	+	2978.23
38	Redox-sensitive transcriptional activator SoxR	WP_003820813.1	Chain A, Redox-sensitive transcriptional activator SoxR [*Escherichia coli* K-12]	2ZHG_A	92%	57.75%	92	58	+	32.55

**Expressions have reported based on the transcript per million (TPM) of RNA-seq data.

### 3.2 Shortlisted promising immunogenic proteins as potential vaccine candidates

A schematic illustration of the stages involved in vaccine target identification is presented in Figure 1B. All 128 non-redundant proteins were retrieved from three sources, including the core proteome of 554 completed genomes, total protein from the *B. pertussis* strain CS, and 11 confirmed virulence factors. In the next step, several consecutive analyses were performed, including antigenicity, allergenicity, prevalence, PSI-BLAST (checking human sequence similarity), and the determination of protein roles. In total, 76 proteins were considered for further analysis (see the Supplementary Table S6). Immunoinformatics evaluations were able to detect potential epitopes, including linear and conformational B-cell and T-cell epitopes. The quartile scoring method showed that the nine proteins were potentially immunogenic. The best immunogenic targets, were considered as potential vaccine candidates, including four autotransporter proteins, namely BapA (WP_010930805.1), BrkA (WP_010931506.1), SphB2 (WP_041166323.1), and TcfA (WP_010930243.1), three adhesion proteins, namely hook-filament junction FliK (WP_041166144.1), fibril protein (WP_010930199.1), and TolA (WP_010931418.1). In addition, a lipoprotein with enzymatic activity including peptidoglycan DD-metalloendopeptidase (WP_003811022.1) and an I78 family peptidase inhibitor protein (WP_003812179.1) showed promising immunogenic targets. See Table 2. High-scoring conformational epitopes (ElliPro tool, threshold ≥0.8) are highlighted in different colors on the proteins. See Figure 2B. Detailed information about the quartile scoring system is provided in the Supplementary Table S7.

**TABLE 2:** The most promising potential immunogenic candidates against *B. pertussis*. The consecutive analyses, B-cell epitope ratios, MHC-II ratios and quartile scores of the protein have been shown.

**No.**	Protein	Subcellular localization	Transmembrane helices	Antigenicity	Allegenecity	Human Blast	VICMpred	EGGNOG5	CD domain search	Prevalence (%)	MW (KDa)	B-cell epitope ratio	MHC II ratio	Quartile score	Expression^*^
1	SphB2 (WP_041166323.1)	Outer Membrane 7.00	Outside	1.1930 (Probable ANTIGEN)	Non-Allergen (0.12)	BLAST (-)	Information and storage (-0.870)	Outer membrane Autotransporter Barrel domain	Uncharacterized conserved protein, contains a C-terminal beta-barrel porin domain [Function unknown]	99.56	98	0.459	1.97	12	54.15
2	TcfA (WP_010930243.1)	Outer Membrane (This protein may have multiple localization sites.) 10.00	Outside	0.9705 (Probable ANTIGEN)	Non-Allergen (0.08)	BLAST (-)	Metabolism Molecule (0.635)	Outer membrane Autotransporter	Outer membrane autotransporter barrel domain	100	66	0.378	2.296	10	5069.80
3	TolA (WP_010931418.1)	Outer Membrane 7.00	Outside	0.9508 (Probable ANTIGEN)	Non-Allergen (0.23)	BLAST (-)	Cellular process (1.616)	Cell envelope Biogenesis protein TolA	TolA protein ;TolA couples the inner membrane complex of itself with TolQ and TolR to the outer membrane complex of TolB and OprL (also called Pal).	100	35	0.354	2.021	10	279.49
4	I78 family peptidase inhibitor protein (WP_003812179.1)	Extracellular 9.65	Outside	0.8856 (Probable ANTIGEN)	Non-Allergen (0.19)	BLAST (-)	Cellular process (1.955)	Peptidase inhibitor I78 family	Peptidase inhibitor I78 family; This family includes Aspergillus elastase inhibitor and belongs to MEROPS peptidase inhibitor family I78.	99.78	11	0.175	2.026	9	307.17
5	Peptidoglycan DD-metalloendopeptidase (WP_003811022.1)	Outer Membrane 8.86	Outside	0.7385 (Probable ANTIGEN)	Non-Allergen (-0.24)	BLAST (-)	Metabolism Molecule (0.636)	Membrane proteins related to metalloendopeptidases	Murein hydrolase activator NlpD	100	30	0.306	1.697	8	667.15
6	Flagellar hook-length control protein FliK (WP_041166144.1)	Extracellular 9.71	Outside	0.7489 (Probable ANTIGEN)	Non-Allergen (-0.19)	BLAST (-)	Cellular process (0.583)	N/D	C-terminal domain of type III secretion proteins FliK, HrpP, YscP, and similar domains	100	33	0.523	1.507	8	27.65
7	Fimbrial protein (WP_010930199.1)	Extracellular 10.00 Secondary localization(s): Fimbrial	Outside	0.8217 (Probable ANTIGEN)	Non-Allergen (-0.36)	BLAST (-)	Virulence factors (0.951)	Cell adhesion	Fimbrial protein	100	22	0.28	1.638	8	2200.34
8	BapA (WP_010930805.1)	Outer Membrane (This protein may have multiple localization sites.) 10.00	Outside	0.7688 (Probable ANTIGEN)	Non-Allergen (0.37)	BLAST (-)	Information and storage (-0.915)	Outer membrane Autotransporter Barrel domain	1. Autotrans_barl: outer membrane autotransporter barrel domain [Protein fate, Protein and peptide secretion and trafficking, Cellular processes, Pathogenesis] 2. PRK15313 super family: intestinal colonization autotransporter adhesin MisL	100	91	0.344	1.665	8	133.97
9	BrkA (WP_010931506.1)	Outer Membrane (This protein may have multiple localization sites.) 10.00	Outside	0.8504 (Probable ANTIGEN)	Non-Allergen (0.08)	BLAST (-)	Information and storage (-0.884)	Outer membrane autotransporter Barrel domain	1. Autotrans_barl: outer membrane autotransporter barrel domain 2. PL1_Passenger_AT: Pertactin-like passenger domains (virulence factors) 3. PRK12688 super family: flagellin	100	103	0.348	1.54	8	1846.86

*Expressions have reported based on the transcript per million (TPM) of RNA-seq data.

### 3.3 Computational confirmation of the MEV and results of *in silico* immune simulation

**FIGURE 3:**
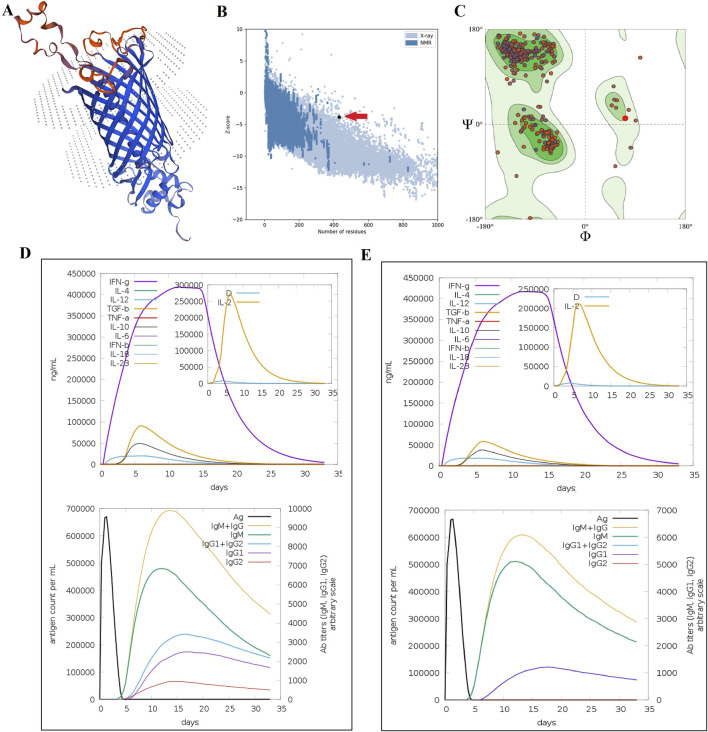
Homology modeling and folding validation of MEVs based on the SphB2 β-barrel domain as an epitope delivery platform. **(A)** Homology modeling of MEVs using the SWISS-MODEL web server. The red regions show the implanted extracellular loops of three autotransporter proteins. **(B)** Model validation of MEV using ProSA-web. The red arrow shows the quality score of protein folding based on the Z-score extracted from crystallography and NMR proteins in the PDB database. **(C)** Ramachandran plot of MEV using the QMEAN model evaluation server. More than 95% of amino acids are located in the acceptable zones of Ramachandran plots. **(D)** *In silico* immune simulation of MEV. **(E)** Immune simulation of intact β-barrel domain of SphB2. The MEV induces a stronger immune response compared to the intact β-barrel domain of SphB2, as seen by higher peak cytokine levels and antibody concentrations in Graph D. Both constructs trigger a Th1-skewed response, marked by the dominance of IFN-γ. However, the MEV leads to a more pronounced Th1 response. The higher levels of IgG1 and IgG2 in the MEV simulation suggest that MEV is more effective in generating a robust and sustained humoral response, which is critical for long-term immunity and vaccine efficacy.

The MEV was designed based on the β-barrel domain of SphB2 as an epitope-delivery platform and six linear B-cell epitopes on the extracellular loops of autotransporter proteins. The homology modeling and folding validation of the MEV were then performed. The SWISS-MODEL results and ProSA analysis showed an acceptable folding of the MEVs. Ramachandran’s plot of MEV showed that more than 95% of the amino acids were in the acceptable zones of the plot. See Figures 3A–C. The criteria for selecting epitopes from the three autotransporter proteins are presented in Table 3. The protein sequences of SphB2 and MEV have been shown in Supplementary Material S8.

**TABLE 3:** Detailed information on antigenicity, allergenicity, conservancy and human proteome BLAST of linear B-cell epitopes that are located on the extracellular loops of the autotransporter proteins of *B. pertussis.* The underlined epitopes were used in MEV.

No.	**Protein**	**Exposed epitopes (extracellular loops)**	Antigenicity	Allergenicity (ML Score)	**Conservancy**	**Human BLAST**
1	**sphB2**	LGLAWQAGAPMAASGGAPGAAALPGAPS	0.6392 (Probable ANTIGEN)	0.35 (Allergen)	82.22% (37/45)	BLAST negative
		**GADTALANGWRVG**	1.0372 (Probable ANTIGEN)	0.29 (Non-Allergen)	84.44% (38/45)	BLAST negative
		RAWDAGAGRLNL	0.8947 (Probable ANTIGEN)	0.33 (Allergen)	84.44% (38/45)	BLAST negative
		YAMPLGMTAELEP	0.7889 (Probable ANTIGEN)	0.36 (Allergen)	82.22% (37/45)	BLAST negative
		**RVAAPLGAGATLRAM**	0.6382 Probable ANTIGEN)	0.22 (Non-Allergen)	84.44% (38/45)	BLAST negative
		GIEVARNAFLDAA	0.2265 (Probable NON-ANTIGEN)	0.33 (Allergen)	84.44% (38/45)	BLAST negative
2	**BrkA**	**GVDAALGKGHNLYA**	1.2847 (Probable ANTIGEN)	0.29 (Non-Allergen)	100.00% (21/21)	BLAST negative
		LRFGRRIALAGGNIVQPY	0.1716 (Probable NON-ANTIGEN)	0.42 (Allergen)	100.00% (21/21)	BLAST negative
		LEGGRRFELPNDWFAEP	0.2898 (Probable NON-ANTIGEN)	0.32 (Allergen)	100.00% (21/21)	BLAST negative
		AAYVGDGGYYLDTVL	-0.3005 (Probable NON-ANTIGEN)	0.34 (Allergen)	100.00% (21/21)	BLAST negative
		LEIGLDRGWSASGGRWYAGGL	0.2183 (Probable NON-ANTIGEN)	0.26 (Non-Allergen)	100.00% (21/21)	BLAST negative
		**LGELRLRADAGGPWA**	0.4428 (Probable ANTIGEN)	0.22 (Non-Allergen)	100.00% (21/21)	BLAST negative
3	**BapA**	GVGADYGWGERYAIYGQ	0.7349 (Probable ANTIGEN)	0.34 (Allergen)	92.31% (12/13)	BLAST negative
		**LDYKSSWQAGGANRESSV**	1.1710 (Probable ANTIGEN)	0.3 (Non-Allergen)	92.31% (12/13)	BLAST negative
		GKAFGLREGLALIP	0.2631 (Probable NON-ANTIGEN)	0.35 (Allergen)	92.31% (12/13)	BLAST negative
		PTLTWYGRDGAYVDAQ	0.2905 (Probable NON-ANTIGEN)	0.34 (Allergen)	76.92% (10/13)	BLAST negative
		**GVDRILAGGQEGSRLVGG**	0.6939 (Probable ANTIGEN)	0.26 (Non-Allergen)	92.31% (12/13)	BLAST negative

The cytokine profile following one injection of MEV revealed high levels of IFN- γ and IL-2, as a result of *in silico* simulations of the behavior of the immune system, carried out on the C-ImmSim platform. Additionally, IgG1 and IgG2 isotypes were increased. In addition, after one injection of the intact β-barrel domain of SphB2, lower levels of IL-2, IgG1, and IgG2 were detected compared with MEV. See Figures 3D,E.

### 3.4 Results of molecular docking and molecular dynamic simulations

**FIGURE 4:**
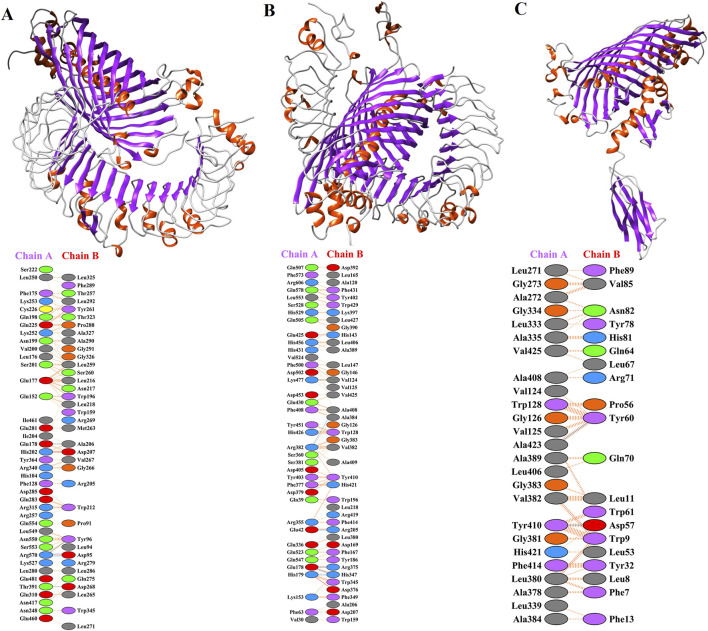
Molecular dockings of MEV with immune receptors including TLR-2, TLR-4, and HLA-DR-B. Residue colors: Blue: Positive (H, K, R); Red: negative (D, E); Green: Neutral (S, T, N, Q); Gray: aliphatic (A, V, L, I, M); Purple: aromatic (F, Y, W); Brown: Pro and Gly; Yellow: Cysteine. Bond colors: Red: Salt bridges; Yellow: Di-sulphide bonds; Blue: Hydrogen bonds; Orange: Non-bonded contacts. The number of H-bond lines between any two residues indicates the number of potential hydrogen bonds between them. For non-bonded contacts, which can be plentiful, the width of the striped line is proportional to the number of atomic contacts. **(A)** The possible interaction of MEV with TLR-2 (PDB ID: 2Z7X_A) and detailed amino acid interactions between TLR2 (chain A) and MEV (chain B). **(B)** The deep lateral interaction of MEV with TLR-4 (PDB ID: 3FXI_A) and the amino acids involved in the interaction have been shown in TLR-4 (chain A) and MEV (chain B). **(C)** The molecular interaction of HLA-DR-B (PDB ID: 3PDO_B) with MEV and the interactions in the amino acid layer have been depicted between MEV (chain A) and HLA-DR-B (chain B).

Molecular docking of MEVs with immune receptors including TLR-2, TLR-4, and HLA-DR-B was performed. The total binding energies of TLR-2, TLR-4, and HLA-DR-B with MEV were −28.374, −78.266, and −68.122, respectively. In the molecular docking process, a more negative total energy indicates greater binding affinity and stability of the complex. The detailed amino acid interactions between immune receptors and MEV are depicted in Figures 4A–C.

**FIGURE 5:**
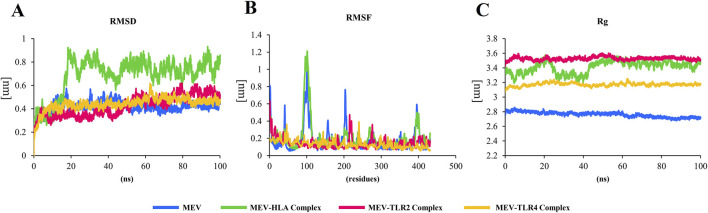
**(A)** In all complexes, the RMSD plot remains below 1, indicating the stability of the structure. The MEV-TLR2 and MEV-TLR4 complexes exhibit fewer fluctuations, suggesting that these complexes are more stable. **(B)** The RMSF values are also <1, but in the sole MEV and the MEV-HLA complex, there are slightly higher fluctuations in residues 96 to 103. However, this region is not involved in receptor-ligand interactions. The binding of TLR2 and TLR4 to MEV reduces the fluctuations of amino acids, making these complexes more stable compared to the MEV alone. **(C)** The Rg plot shows minimal fluctuations, indicating that the complexes are stable. The higher Rg observed in the complexes compared to the protein alone is due to the binding of the protein to ligands, which leads to a larger complex, increased radius of gyration, and lower molecular structural compactness.

MD simulations were conducted to determine the feasibility of the interactions in docked complexes. The RMSD, RMSF, and Rg are key parameters used in MD simulations. The RMSD is used to assess the overall stability of a biomolecular system by measuring how much the structure deviates over time (Nipun et al., 2021). In all complexes, the RMSD values remain below 1, indicating structural stability. The MEV-TLR2 and MEV-TLR4 complexes exhibit fewer fluctuations, suggesting that these complexes are more stable (Figure 5A). The RMSF provides insights into the flexibility of individual residues within the molecule (Nipun et al., 2021). The RMSF values are also <1, but in the sole MEV and the MEV-HLA- DR-B complex, there are slightly higher fluctuations in residues 96 to 103. However, this region does not participate in receptor-ligand interactions. The predicted binding of TLR2 and TLR4 to MEV reduces the fluctuations of amino acids, making these complexes more stable compared to the MEV alone (Figure 5B). Rg highlights the compactness of the structure by measuring the distribution of atoms around the center of mass (Nipun et al., 2021). In our analysis, the Rg plot shows minimal fluctuations, indicating that the complexes are stable. The higher Rg observed in the complexes compared to the protein alone results from ligand binding, which increases complex size, radius of gyration, and decreases structural compactness (Figure 5C).

## 4 Discussion

Pertussis remains a major cause of morbidity and mortality in infants worldwide, despite high vaccination rates (Noel et al., 2020). The resurgence of this disease necessitates the development of new generations of vaccines and strategies for prevention. There are only a few studies on genomics and reverse vaccinology approaches to introduce new vaccine candidates and novel drug targets. In a study by Felice et al., comparative genomics analyses of 20 genomes were performed. This study proposed five drug targets (WlbB, CheY, BP1651, DapD and KdsA) and seven vaccine candidates, including Fim3, BfrE, BP2851, BP2219, Vag8, BP3761, and OmpQ (Felice et al., 2022). The new vaccine candidates were mainly secreted proteins and were selected based on adhesion probability scores. Another subtraction genomics-based approach was applied to the *B. pertussis* strain B1917 to identify four novel drug targets, including neuraminidase, Ig heavy chain V-A2 region BS-1, MHC HLA-DC3-beta, and MHC H2-IA-alpha (Jamal et al., 2022). An investigation applied PanRV software to detect 15 vaccine candidates from the core proteome of 603 genomes of *B. pertussis* (Monterrubio-López et al., 2024). Based on the results of all these studies, none of the previous proposed vaccine candidates or drug targets are found in our shortlisted protein.

The differences observed between our findings and those reported in previous reverse vaccinology studies can be attributed to both methodological and biological factors. One major source of variation lies in the diversity of bioinformatics tools and prediction pipelines used across studies. Differences in algorithms, scoring systems, and prioritization strategies can lead to the identification of distinct sets of candidate proteins. Furthermore, the selection of threshold values such as sequence identity, coverage, antigenicity, and subcellular localization can significantly influence the outcomes. In our study, more stringent and updated criteria were applied to enhance the specificity of predicted targets. Additionally, we utilized the most recent genome annotations available for *B. pertussis*, which may differ from those used in earlier studies. Biological factors such as strain variation and genomic updates could also contribute to the observed lack of overlap. Taken together, these methodological and biological variables likely explain the discrepancies and underscore the importance of standardizing computational approaches in reverse vaccinology research.

Macrolide-resistant *B. pertussis* has been increasingly reported in China during the past decade, raising concerns about its potential transmission to other regions and countries (Feng et al., 2021; Ivaska et al., 2022b). On the other hand, frequent use of antibiotics e.g., azithromycin in COVID-19 pandemic threatens to intensify antimicrobial resistance all over the world (Langford et al., 2023; Antonazzo et al., 2022). The introduction of novel drug targets against pertussis appears worthwhile given the limited available drugs of choice to treat this infection.

Structure-based virtual screening (SBVS) is a useful and promising *in silico* strategy for drug design (da Rocha et al., 2024). A prerequisite for SBVS is the availability of a tertiary structure of the target drug protein. However, homology modeling allows us to predict the 3D structure of a protein based on its homology in the Protein Data Bank. In this study, we identified 38 feasible and novel drug targets against *B. pertussis*. These essential proteins have significant homology with 3D characterized proteins deposited in PDB. These proteins are involved in replication, transcription, translation, and metabolic pathways in bacteria. It appears that these proteins are promising drug targets for SBVS. Thus, further studies are required to identify potential inhibitors against the introduced drug targets.

We acknowledge the limitations of structure-based virtual screening (SBVS) in targeting ribosomal proteins due to the structural complexity of bacterial ribosomes and the intricate interactions among ribonucleoprotein components (Zhang et al., 2021). While our initial analysis identified 14 ribosomal proteins as potential drug targets, we think that pharmacophore modeling based on known ribosome-targeting antibiotics such as those acting on the 30S and 50S subunits offers a more practical and biologically feasible approach (CP et al., 2019). Additionally, targeting ribosome-associated factors, such as hibernation factors, may provide more specific and accessible alternatives (Ekemezie and Melnikov, 2024). Emerging structural biology techniques, particularly cryo-electron microscopy (cryo-EM), have significantly advanced our understanding of ribosomal architecture and dynamics, enabling more accurate structure-guided drug design (Wang et al., 2023). Incorporating these approaches represents a promising direction for improving the selectivity and efficacy of antibacterial drug discovery targeting ribosomal machinery.

The resurgence of pertussis and its drawbacks with current vaccines has prompted researchers to consider the introduction of new candidates in pertussis vaccine formulation. To identify new vaccine targets, a deep understanding of host-pathogen interaction is necessary. New technologies and strategies can be used to characterize novel vaccine targets. Immunoproteomics and transposon-directed insertion sequencing identified 13 factors, including BP0840 (OmpP), Pal, OmpA2, BP1485, BamA, Pcp, MlaA, YfgL, BP2197, BP1569, MlaD, ComL, and BP0183 (Gregg et al., 2023). These antigens are identified as immunogenic and essential and can be considered in vaccine formulations to reduce or deprive vaccinated individuals of bacteria. In addition, immunology-based studies could potentially shed light on the pertussis vaccine failure, potentially leading to strategies for reversing this trend. *In vivo* studies have provided evidence that respiratory CD4^+^ tissue-resident memory T (T_RM_) cells that release IL-17 and IFN-γ have an important role in conferring immunity against *B. pertussis* nasal infection (Mills, 2023). Furthermore, nasal delivery of vaccines can help with this process. Live attenuated vaccines and mucosal immunity-inducing adjuvants along with recombinant subunit vaccine candidates are two strategies for producing a new generation of pertussis vaccines (Lavelle and Ward, 2022; Solans and Locht, 2019).

In this study, nine shortlisted proteins were chosen. Four autotransporter proteins, namely BapA, BrkA, SphB2, and TcfA, demonstrated significant potential as vaccine candidates. Thus, BrkA and SphB2 were selected for vaccine formulations. Investigations have highlighted that the autotransporter BrkA does not confer lung protection by itself but increases lung protection in combination with pertussis toxin and filamentous hemagglutinin (Marr et al., 2008). Other autotransporter, such as SphB1 and SphB2, were also evaluated as protective antigens against colonization by *B. pertussis* in the respiratory tract (Suzuki et al., 2017). One patent (Google patents: WO2005032584A2) applied BapA in combination with other vaccine candidates. However, TcfA was not assessed in any experimental study. The flagellar hook-length control protein FliK (involved in secretion of flagellar FliK protein) has not been used in vaccine formulation; however, fimbrial proteins are classic components of pertussis vaccines (Xu et al., 2009). Peptidoglycan DD-metalloendopeptidase (NlpD super family protein) links cell wall remodeling and outer membrane invagination during cell separation in *B. pertussis* (Tsang et al., 2017; Stohl et al., 2016). Although there are no reports on the use of this protein in immunization against pertussis, it was used in vaccine formulations for *Yersinia pestis* and *Neisseria gonorrhea* (Sun and Singh, 2019; Semchenko and Seib, 2022). Based on the STRING database, the TolA protein (WP_010931418.1) may be involved in protein import and the integrity of the outer membrane. The peptidase inhibitor I78 family protein (WP_003812179.1) is an uncharacterized protein in *B. pertussis* that interacts with several hypothetical proteins. However, this factor is involved in the pathogenesis of *Acinetobacter baumannii* (Zou et al., 2023). Homology modeling was unsuccessful for the flagellar hook-length control proteins FliK, PD-metalloendopeptidase, TcfA, and TolA due to the absence of homologous PDB files in the Protein Data Bank. Taken together, this study suggests that autotransporter proteins are the most promising vaccine candidates.

It seems that the use of RNA-seq has a very important role in finding novel drug targets and immunogenic candidates. It is assumed that genes with higher expression are more important for bacterial physiology. For example, rRNAs and ribosomal proteins have the highest expression. In addition, subtractive analysis of RNA-seq in different conditions can facilitate the detection of key factors in bacterial pathogenesis. In this study, RNA-seq analysis of CS strain showed that some proteins including CheZ and CheW are not expressed and may be not suitable factors for medicinal purposes. On the other hand, the low expression of proteins such as FliK and SphB2 in the list of immunogenic candidates should be interpreted with more caution and used in vaccine formulations.

Multi-epitope vaccines (MEV) have resulted from advancements in immunoinformatics analysis and protein engineering. MEVs are used to treat cancer and infectious diseases (Zhang, 2018). Platforms that carry or deliver immunogenic epitopes to the immune system can be protein scaffolds. Immune-reactive domains of other proteins can also be used as adjuvants in MEVs. In this study, we used the β-barrel domain of SphB2 as a MEV platform. This domain has six extracellular loops as disorder regions for implanting foreign epitopes from other immunogenic targets. Note that the extracellular loops of the β-barrel domain in outer membrane proteins play critical roles in the pathogenesis and host-cell interactions of bacteria (Magaña et al., 2023). We collected immunogenic and conserved epitopes of extracellular loops from autotransporter proteins. The results of immune simulation and molecular interactions are promising. Molecular dockings and dynamic simulations showed that MEV interacts significantly with TLR-2, TLR-4, and HLA-DR receptors. However, the cytokine profiles and rise in the different isotypes of antibodies revealed that this MEV needs a helper adjuvant such as monophosphoryl lipid A (MPL) to boost the immune system toward Th1 response in experimental assays in the future of this investigation. A limitation of this study is the lack of experimental validation to support the findings. The study’s conclusions are based exclusively on *in silico* analysis using subtractive genomics and the reverse vaccinology method.

## 5 Conclusion

It appears that the resurgence of pertussis requires new vaccine candidates, novel drug targets, and modern strategies to deal with this highly contagious bacterium. No doubt, the evolutionary mechanisms of the *B. pertussis* genome will continue, and this is a remarkable struggle between humans and bacteria. Despite the genomics revolution and the use of bioinformatics tools to invent and propose suitable targets for vaccines and drug targets, time will take place in favor of humankind. The results of this study highlighted autotransporter proteins as potential immunogenic targets. It seems that the pertussis vaccine needs an updated list of different candidates to prevent resurgence. This study has proposed some potential candidates. Moreover, MEV designs using immunoinformatics appear to be a new generation of vaccine candidates. In addition, proteins involved in translation and metabolic pathways can be considered novel drug targets. Different cocktails of vaccine candidates, the use of appropriate adjuvants to stimulate the immune system for protection, and the development of new drugs against new therapeutic targets can help prevent the resurgence of pertussis. Undoubtedly, experimental validation is necessary to confirm the findings of this study.

## Data Availability

The original contributions presented in the study are included in the article/Supplementary Material, further inquiries can be directed to the corresponding author.
