# Supplementary material for: Exploiting subtractive genomics to identify novel drug targets and new immunogenic candidates against Bordetella pertussis: an in silico study
Source: Front Bioinform. 2025 May 13;5:1570054. doi: 10.3389/fbinf.2025.1570054 (PMC12106433; doi:10.3389/fbinf.2025.1570054)
Supplement: Supplementary file 2 [file DataSheet7.docx]

**Supplementary Data S7**. The FASTA file of MEV includes six B-cell epitopes that were implanted in extracellular loops of the beta-barrel domain of SphB2.

>multiepitope_vaccine based on Beta barrel domain of SphB2

GSGDLAFADLADTANQRATARAVETLPAAHPVYRAIETLPEGAPPAAFDALSGEVHASTRSALLTGARQAQRVNLGRLRDHLGLAWQAGAPMALDYKSSWQAGGANRESSVALPGAPSPAWAEVVGAWQRLDGDGNAAQVRQHVGGLFVGADTALANGWRVGGSLGFTDGRIEVDARASQTDVASYTAALYGGRAWDALGELRLRADAGGPWAGRLNLLAGAAYTWHDIASERRVDVGDLRQELTADYGASTTQLFTELSYAMPLGVDRILAGGQEGSRLVGGAELEPFAGLAWNQLRVRGFTETGGSAALSGASSRDDMAITTLGARVAAPLGAGATLRAMAGWRHAFGDRTPQSTLAFGQGSAFEVAGAPIARDAALLGVGAGIEVAGVDAALGKGHNLYAAFLDAAYAGEFGGGNRQHTASVVLRWRF
