# Supplementary material for: Exploiting subtractive genomics to identify novel drug targets and new immunogenic candidates against Bordetella pertussis: an in silico study
Source: Front Bioinform. 2025 May 13;5:1570054. doi: 10.3389/fbinf.2025.1570054 (PMC12106433; doi:10.3389/fbinf.2025.1570054)
Supplement: Supplementary file 3 [file DataSheet6.docx]

**Table S6**. Data on consecutive analysis of the 76 surface-exposed proteins of *B. pertussis* to identify novel vaccine candidates.

| **No.** | **Protein** | **Subcellular localization** | **Transmembrane helices** | **Antigenicity** | **Allegenecity** | **Hybrid score** | **Human Blast** | **VICMpred** | **EGGNOG5** | **CD search** | **prevalence** | **MW (KDa)** | **B-cell epitopes ratio** | **MHC II ratio** |
| --- | --- | --- | --- | --- | --- | --- | --- | --- | --- | --- | --- | --- | --- | --- |
| 1 | fimB | Cellwall 3.33  Extracellular 3.33 | Outside | 0.6433 (Ag) | Non-Allergen | -0.46 | BLAST_ | Metabolism Molecule (1.793) | pilus organization | pilus assembly protein | 100 | 26 | 0.008 | 1.504 |
| 2 | NZ_CP086368_CDS_LMF30_RS00840 | OuterMembrane 10.00 | Outside | 0.6551 (Ag) | Non-Allergen | 0.15 | BLAST_ | Virulence factors (0.725) | TonB-dependent receptor | 1. TonB-hemin super family: TonB-dependent heme/hemoglobin receptor family protein  2. STN:  Secretin and TonB N terminus short domain | 100 | 94 | 0.183 | 1.19 |
| 3 | NZ_CP086368_CDS_LMF30_RS01660 | OuterMembrane 10.00 | Outside | 0.7084 (Ag) | Non-Allergen | 0.01 | BLAST_ | Metabolism Molecule (0.215) | membrane organization | Outer membrane translocation and assembly module TamA | 100 | 68 | 0.048 | 1.018 |
| 4 | NZ_CP086368_CDS_LMF30_RS02925 | OuterMembrane 10.00 | Outside | 0.6853 (Ag) | Non-Allergen | -0.42 | BLAST_ | Cellular process (-0.712) | Outer membrane efflux protein | efflux transporter, outer membrane factor (OMF) lipoprotein, NodT family | 100 | 53 | 0.086 | 0.903 |
| 5 | NZ_CP086368_CDS_ptxA | Extracellular 10.00 | Outside | 0.5463 (Ag) | Non-Allergen | 0.28 | BLAST_ | Metabolism Molecule (2.784) | NAD+ ADP-ribosyltransferase activity | Pertussis toxin, subunit | 99.67 | 29 | 0.141 | 0.981 |
| 6 | NZ_CP086368_CDS_LMF30_RS03215 | OuterMembrane 10.00 | Outside | 0.8942 (Ag) | Non-Allergen | 0.12 | BLAST_ | Cellular process (2.411) | Outer membrane protein beta-barrel domain | Porin superfamily. These outer membrane channels share a beta-barrel structure that differ in strand and shear number | 100 | 23 | 0.08 | 0.962 |
| 7 | NZ_CP086368_CDS_LMF30_RS03675 | OuterMembrane 10.00 | Outside | 0.5855 (Ag) | Non-Allergen | -0.29 | BLAST_ | Cellular process (2.139) | Outer membrane protein beta-barrel domain | Porin superfamily. These outer membrane channels share a beta-barrel structure that differ in strand and shear number. | 100 | 79 | 0.108 | 1.278 |
| 8 | NZ_CP086368_CDS_brkA | OuterMembrane (This protein may have multiple localization sites.) 10.00 | Outside | 0.8504 (Ag) | Non-Allergen | 0.08 | BLAST_ | Information and storage (-0.884) | outer membrane autotransporter barrel | 1. autotrans_barl: outer membrane autotransporter barrel domain  2. PL1_Passenger_AT: Pertactin-like passenger domains (virulence factors)  3. PRK12688 super family:  flagellin | 100 | 103 | 0.348 | 1.54 |
| 9 | NZ_CP086368_CDS_LMF30_RS04990 | OuterMembrane 10.00 | Outside | 0.6399 (Ag) | Non-Allergen | -0.24 | BLAST_ | Virulence factors (1.007) | Outer membrane protein (Porin) | Porin superfamily. These outer membrane channels share a beta-barrel structure that differ in strand and shear number. | 100 | 39 | 0.178 | 1.782 |
| 10 | NZ_CP086368_CDS_LMF30_RS05180 | OuterMembrane 9.52 | Outside | 0.5975 (Ag) | Non-Allergen | 0.04 | BLAST_ | Information and storage (-0.885) | Phage-related minor tail protein | 1. TMP_2: Prophage tail length tape measure protein; This family represents a conserved region located towards the N-terminal end of prophage tail length tape measure protein (TMP). TMP is important for assembly of phage tails and involved in tail length determination. Mutated forms TMP cause tail fibers to be shortened.  2. COG5281 super family: Prophage tail length tape measure protein ;This family represents a conserved region located towards the N-terminal end of prophage tail length tape measure protein (TMP). TMP is important for assembly of phage tails and involved in tail length determination. Mutated forms TMP cause tail fibers to be shortened. | 99.67 | 92 | 0.044 | 1.349 |
| 11 | NZ_CP086368_CDS_tolA | OuterMembrane 7.00 | Outside | 0.9508 (Ag) | Non-Allergen | 0.23 | BLAST_ | Cellular process (1.616) | Cell envelope biogenesis protein TolA | TolA protein ;TolA couples the inner membrane complex of itself with TolQ and TolR to the outer membrane complex of TolB and OprL (also called Pal). | 100 | 35 | 0.354 | 2.021 |
| 12 | NZ_CP086368_CDS_pal | OuterMembrane 10.00 | Outside | 0.7531 (Ag) | Non-Allergen | 0.13 | BLAST_ | Cellular process (1.097) | Belongs to the ompA family | peptidoglycan-associated lipoprotein; Members of this protein are Pal (also called OprL), the Peptidoglycan-Associated Lipoprotein of the Tol-Pal system. The system appears to be involved both in the maintenance of outer membrane integrity and in the import of certain organic molecules as nutrients. | 100 | 17 | 0.139 | 1.357 |
| 13 | NZ_CP086368_CDS_LMF30_RS05365 | OuterMembrane 10.00 | Outside | 0.5624 (Ag) | Non-Allergen | 0.21 | BLAST_ | Cellular process (1.080) | lipopolysaccharide transport | LPS assembly outer membrane protein LptD (organic solvent tolerance protein OstA) [Cell wall/membrane/envelope biogenesis] | 100 | 88 | 0.112 | 1.581 |
| 14 | NZ_CP086368_CDS_LMF30_RS05390 | OuterMembrane 9.92 | Outside | 0.8135 (Ag) | Non-Allergen | 0.17 | BLAST_ | Cellular process (1.436) | Belongs to the ompA family | Peptidoglycan binding domains similar to the C-terminal domain of outer-membrane protein OmpA ;OmpA-like domains | 99.67 | 21 | 0.085 | 1.615 |
| 15 | NZ_CP086368_CDS_LMF30_RS05475 | OuterMembrane 10.00 | Outside | 0.6218 (Ag) | Non-Allergen | 0.26 | BLAST_ | Cellular process (1.403) | Outer membrane receptor | 1. PRK10044 super family: ferrichrome outer membrane transporter  2. STN: Secretin and TonB N-terminus short domain in type II/III secretory system | 100 | 89 | 0.097 | 1.361 |
| 16 | NZ_CP086368_CDS_cyaE | OuterMembrane 10.00 | Outside | 0.6320 (Ag) | Non-Allergen | -0.43 | BLAST_ | Information and storage (-0.727) | outer membrane efflux protein | Outer membrane protein TolC [Cell wall/membrane/envelope biogenesis] | 100 | 50 | 0.05 | 1.246 |
| 17 | NZ_CP086368_CDS_LMF30_RS05675 | OuterMembrane 7.00 | Outside | 0.6141 (Ag) | Non-Allergen | 0.06 | BLAST_ | Metabolism Molecule (0.208) | Autotransporter beta-domain | Autotransporter beta-domain. This domain is found at the C-terminus of the proteins it occurs in. | 99.67 | 35 | 0.055 | 1.45 |
| 18 | NZ_CP086368_CDS_LMF30_RS06000 | OuterMembrane 10.00 | Outside | 0.6039 (Ag) | Non-Allergen | -0.16 | BLAST_ | Cellular process (2.348) | Gram-negative porin | Porins form aqueous non-specific channels for the diffusion of small hydrophillic molecules across the outer membrane. | 100 | 41 | 0.2 | 1.66 |
| 19 | NZ_CP086368_CDS_LMF30_RS06080 | OuterMembrane 10.00 | Outside | 0.6377 (Ag) | Non-Allergen | -0.35 | BLAST_ | Cellular process (1.853) | Receptor | Outer membrane receptor for monomeric catechols [Inorganic ion transport and metabolism] | 100 | 81 | 0.11 | 1.613 |
| 20 | NZ_CP086368_CDS_LMF30_RS06085 | OuterMembrane 9.95 | Outside | 0.6575 (Ag) | Non-Allergen | -0.12 | BLAST_ | Virulence factors (2.022) | Receptor | Outer membrane receptor for monomeric catechols [Inorganic ion transport and metabolism] | 100 | 81 | 0.138 | 1.554 |
| 21 | NZ_CP086368_CDS_LMF30_RS06190 | OuterMembrane 9.52 | Outside | 0.6865 (Ag) | Non-Allergen | 0.19 | BLAST_ | Virulence factors (1.853) | 1-acyl-2-lysophosphatidylserine acylhydrolase activity | The outer membrane phospholipase A (OMPLA) is an integral membrane enzyme that catalyses the hydrolysis of acylester bonds in phospholipids using calcium as a cofactor. involved in pathogenesis and virulence. | 100 | 47 | 0.094 | 1.618 |
| 22 | NZ_CP086368_CDS_LMF30_RS06735 | OuterMembrane (This protein may have multiple localization sites.) 9.95 | Outside | 0.5640 (Ag) | Non-Allergen | -0.36 | BLAST_ | Cellular process (1.493) | RND efflux system, outer membrane lipoprotein | Cu(I)/Ag(I) efflux RND transporter outer membrane protein | 100 | 33 | 0.049 | 1.427 |
| 23 | NZ_CP086368_CDS_LMF30_RS06785 | Final Prediction:  Extracellular 10.00  Secondary localization(s):  Flagellar | Outside | 0.6965 (Ag) | Non-Allergen | 0.24 | BLAST_ | Cellular process (1.322) | bacterial-type flagellum-dependent cell motility | PRK06819, FliC/FljB family flagellin | 100 | 40 | 0.319 | 1.296 |
| 24 | NZ_CP086368_CDS_prn | OuterMembrane (This protein may have multiple localization sites.) 10.00 | Outside | 0.7505 (Ag) | Non-Allergen | -0.41 | BLAST_ | Virulence factors (-0.470) | Outer membrane autotransporter | 1. autotrans_barl: outer membrane autotransporter barrel domain; A number of Gram-negative bacterial proteins, mostly found in pathogens and associated with virulence  2. PL1_Passenger_AT: Pertactin-like passenger domains (virulence factors), C-terminal, subgroup 1, of autotransporter proteins of the type V secretion system of Gram-negative bacteria. | 100 | 93 | 0.194 | 1.473 |
| 25 | NZ_CP086368_CDS_LMF30_RS07435 | Final Prediction:  Extracellular 10.00  Secondary localization(s):  Fimbrial | Outside | 0.8217 (Ag) | Non-Allergen | -0.36 | BLAST_ | Virulence factors (0.951) | cell adhesion | Fimbrial protein | 100 | 22 | 0.28 | 1.638 |
| 26 | NZ_CP086368_CDS_LMF30_RS07530 | OuterMembrane 10.00 | Outside | 0.6577 (Ag) | Non-Allergen | 0.14 | BLAST_ | Virulence factors  (-0.552) | TonB-dependent siderophore receptor | 1. PRK10044 super family: ferrichrome outer membrane transporter  2. STN: Secretin and TonB N terminus short domain | 2.83 | 89 | 0.117 | 1.607 |
| 27 | NZ_CP086368_CDS_LMF30_RS07790 | OuterMembrane 9.93 | Outside | 0.5169 (Ag) | Non-Allergen | 0.12 | BLAST_ | Metabolism Molecule (1.042) | NlpE N-terminal domain | NlpE N-terminal domain representing a bacterial outer membrane lipoprotein that is necessary for signalling by the Cpx pathway | 100 | 15 | 0.133 | 1.943 |
| 28 | NZ_CP086368_CDS_tcfA | OuterMembrane (This protein may have multiple localization sites.) 10.00 | Outside | 0.9705 (Ag) | Non-Allergen | 0.08 | BLAST_ | Metabolism Molecule (0.635) | Outer membrane autotransporter | outer membrane autotransporter barrel domain | 100 | 66 | 0.378 | 2.296 |
| 29 | NZ_CP086368_CDS_LMF30_RS08585 | OuterMembrane (This protein may have multiple localization sites.) 9.83 | Outside | 0.8224 (Ag) | Non-Allergen | 0.24 | BLAST_ | Information and storage (-0.881) | outer membrane autotransporter | outer membrane autotransporter barrel domain | 100 | 88 | 0.321 | 1.496 |
| 30 | NZ_CP086368_CDS_flgB | Periplasmic 9.44  Secondary localization(s):  Flagellar | Outside | 0.6889 (Ag) | Non-Allergen | -0.4 | BLAST_ | Cellular process (1.167) | Flagellar basal body rod | flagellar basal body rod protein FlgB | 99.89 | 15 | 0.222 | 1.222 |
| 31 | NZ_CP086368_CDS_LMF30_RS08735 | Extracellular 9.72  Secondary localization(s):  Fimbrial | Outside | 0.5602 (Ag) | Non-Allergen | -0.3 | BLAST_ | Metabolism Molecule (1.366) | Required for flagellar hook formation. May act as a scaffolding protein | FlgD Tudor-like domain | 99.89 | 24 | 0.149 | 1.589 |
| 32 | NZ_CP086368_CDS_LMF30_RS08740 | Extracellular 10.00 | Outside | 0.6540 (Ag) | Non-Allergen | 0.35 | BLAST_ | Cellular process (0.773) | flagellar hook-associated protein | flagellar hook protein FlgE | 99.89 | 49 | 0.147 | 1.748 |
| 33 | NZ_CP086368_CDS_flgG | Extracellular 9.71  Secondary localization(s):  Flagellar | Outside | 0.6809 (Ag) | Non-Allergen | -0.28 | BLAST_ | Virulence factors (1.470) | Flagellar basal body rod | flagellar basal body rod protein FlgG | 99.89 | 27 | 0.157 | 1.463 |
| 34 | NZ_CP086368_CDS_LMF30_RS08755 | OuterMembrane 8.86  Secondary localization(s):  Flagellar | Outside | 0.6527 (Ag) | Non-Allergen | -0.27 | BLAST_ | Cellular process (0.446) | Assembles around the rod to form the L-ring and probably protects the motor basal body from shearing forces during rotation | Flagellar L-ring protein | 99.89 | 24 | 0.24 | 1.052 |
| 35 | NZ_CP086368_CDS_LMF30_RS08760 | Periplasmic 9.76  Secondary localization(s):  Flagellar | Outside | 0.5320 (Ag) | Non-Allergen | -0.33 | BLAST_ | Cellular process (2.913) | bacterial-type flagellum-dependent cell motility | Flagellar P-ring protein | 99.89 | 39 | 0.029 | 1.257 |
| 36 | NZ_CP086368_CDS_flgL | Extracellular 9.96  Secondary localization(s):  Flagellar | Outside | 0.6248 (Ag) | Non-Allergen | 0.19 | BLAST_ | Virulence factors (2.650) | bacterial-type flagellum-dependent cell motility | flagellar hook-associated protein FlgL | 99.89 | 53 | 0.198 | 1.501 |
| 37 | NZ_CP086368_CDS_fliM | CytoplasmicMembrane 7.88  Secondary localization(s):  Flagellar | Outside | 0.5809 (Ag) | Non-Allergen | 0.05 | BLAST_ | Cellular process (1.715) | FliM is one of three proteins (FliG, FliN, FliM) that forms the rotor-mounted switch complex (C ring), located at the base of the basal body. This complex interacts with the CheY and CheZ chemotaxis proteins, in addition to contacting components of the motor that determine the direction of flagellar rotation | flagellar motor switch protein FliM | 100 | 38 | 0.107 | 1.586 |
| 38 | NZ_CP086368_CDS_fliL | CytoplasmicMembrane 10.00  Secondary localization(s):  Flagellar | Outside | 0.5768 (Ag) | Non-Allergen | 0.16 | BLAST_ | Cellular process (2.561) | Controls the rotational direction of flagella during chemotaxis | Flagellar basal body-associated protein FliL | 100 | 20 | 0.11 | 1.921 |
| 39 | NZ_CP086368_CDS_LMF30_RS19935 | Extracellular 9.71 | Outside | 0.7489 (Ag) | Non-Allergen | -0.19 | BLAST_ | Cellular process (0.583) | N/D | C-terminal domain of type III secretion proteins FliK, HrpP, YscP, and similar domains | 0 hit from BacMap | 33 | 0.523 | 1.507 |
| 40 | NZ_CP086368_CDS_fliE | Periplasmic 9.44  Secondary localization(s):  Flagellar | Outside | 0.5991 (Ag) | Non-Allergen | -0.32 | BLAST_ | Cellular process (0.413) | Flagellar hook-basal body complex protein FliE | Flagellar hook-basal body complex protein FliE | 100 | 11 | 0.081 | 1.263 |
| 41 | NZ_CP086368_CDS_fliD | Extracellular 9.71  Secondary localization(s):  Flagellar | Outside | 0.7692 (Ag) | Non-Allergen | -0.2 | BLAST_ | Virulence factors (3.183) | N/D | flagellar capping protein | 100 | 47 | 0.091 | 1.212 |
| 42 | NZ_CP086368_CDS_LMF30_RS09700 | OuterMembrane 8.28 | Outside | 0.5902 (Ag) | Non-Allergen | 0.27 | BLAST_ | Metabolism Molecule (1.062) | iron ion transport | Uncharacterized iron-regulated protein [Function unknown] | 100 | 47 | 0.168 | 1.43 |
| 43 | NZ_CP086368_CDS_LMF30_RS09730 | Extracellular 10.00  Secondary localization(s):  Fimbrial | Outside | 0.7296 (Ag) | Non-Allergen | -0.09 | BLAST_ | Cellular process (1.254) | Fimbrial protein | Fimbrial protein | 100 | 21 | 0.232 | 1.406 |
| 44 | NZ_CP086368_CDS_sphB2 | OuterMembrane 7.00 | Outside | 1.1930 (Ag) | Non-Allergen | 0.12 | BLAST_ | Information and storage (-0.870) | outer membrane autotransporter barrel domain | Uncharacterized conserved protein, contains a C-terminal beta-barrel porin domain [Function unknown] | 99.56 | 98 | 0.459 | 1.97 |
| 45 | NZ_CP086368_CDS_LMF30_RS10475 | OuterMembrane 8.86 | Outside | 0.7385 (Ag) | Non-Allergen | -0.24 | BLAST_ | Metabolism Molecule (0.636) | Membrane proteins related to metalloendopeptidases | murein hydrolase activator NlpD | 100 | 30 | 0.306 | 1.697 |
| 46 | NZ_CP086368_CDS_phg | OuterMembrane (This protein may have multiple localization sites.) 10.00 | Outside | 0.6091 (Ag) | Non-Allergen | -0.43 | BLAST_ | Virulence factors (1.708) | outer membrane autotransporter barrel domain | outer membrane autotransporter barrel domain mostly found in pathogens and associated with virulence, contain a conserved C-terminal domain that integrates into the outer membrane and enables the N-terminal region to be delivered across the membrane. | 100 | 45 | 0.184 | 1.538 |
| 47 | NZ_CP086368_CDS_LMF30_RS11520 | Extracellular 9.65 | Outside | 0.8856 (Ag) | Non-Allergen | 0.19 | BLAST_ | Cellular process (1.955) | Peptidase inhibitor I78 family | Peptidase inhibitor I78 family ;This family includes Aspergillus elastase inhibitor and belongs to MEROPS peptidase inhibitor family I78. | 99.78 | 11 | 0.175 | 2.026 |
| 48 | NZ_CP086368_CDS_LMF30_RS12100 | OuterMembrane 10.00 | Outside | 0.5373 (Ag) | Non-Allergen | 0.16 | BLAST_ | Metabolism Molecule (2.723) | TonB-dependent receptor | TonB dependent/Ligand-Gated channels are created by a monomeric 22 strand (22,24) anti-parallel beta-barrel. | 100 | 78 | 0.114 | 1.501 |
| 49 | NZ_CP086368_CDS_LMF30_RS12200 | OuterMembrane 10.00 | Outside | 0.5032 (Ag) | Non-Allergen | 0.07 | BLAST_ | Cellular process (0.290) | Pilus formation protein N terminal region | Flp pilus assembly protein, secretin CpaC [Intracellular trafficking, secretion, and vesicular transport, Extracellular structures] | 100 | 46 | 0.236 | 1.545 |
| 50 | NZ_CP086368_CDS_LMF30_RS12370 | OuterMembrane 10.00 | Outside | 0.6389 (Ag) | Non-Allergen | -0.33 | BLAST_ | Cellular process (2.503) | Outer membrane receptor proteins, mostly Fe transport | PRK10044, ferrichrome outer membrane transporter | 7.86 | 78 | 0.061 | 1.619 |
| 51 | NZ_CP086368_CDS_pgaA | OuterMembrane 9.52 | Outside | 0.5773 (Ag) | Non-Allergen | 0.18 | BLAST_ | Metabolism Molecule (0.772) | Poly-beta-1,6 N-acetyl-D-glucosamine export porin PgaA | poly-beta-1,6 N-acetyl-D-glucosamine export porin PgaA. The PGA polysaccharide adhesin is a critical determinant of biofilm formation. The conserved C-terminal domain of this outer membrane protein is preceded by a variable number of TPR repeats. | 100 | 73 | 0.021 | 1.452 |
| 52 | NZ_CP086368_CDS_fhaC | OuterMembrane 10.00 | Outside | 0.5986 (Ag) | Non-Allergen | 0.17 | BLAST_ | Virulence factors (2.262) | hemolysin activation secretion protein | 1. ShlB: Haemolysin secretion/activation protein ShlB/FhaC/HecB  2. POTRA_2: POTRA domain, ShlB-type; The POTRA domain (for polypeptide-transport-associated domain) is found towards the N-terminus of ShlB family proteins (pfam03865). ShlB is important in the secretion and activation of the haemolysin ShlA. It has been postulated that the POTRA domain has a chaperone-like function over ShlA; it may fold back into the C-terminal beta-barrel channel.  3. POTRA_3: POTRA domain ;This POTRA domain is found in ShlB-like proteins. | 100 | 64 | 0.113 | 1.452 |
| 53 | NZ_CP086368_CDS_LMF30_RS12780 | Extracellular 9.72  Secondary localization(s):  Fimbrial | Outside | 0.6417 (Ag) | Non-Allergen | 0.33 | BLAST_ | Cellular process (1.056) | Fimbrial protein | Pilin (type 1 fimbria component protein) [Cell motility] | 100 | 40 | 0.098 | 1.898 |
| 54 | NZ_CP086368_CDS_LMF30_RS12785 | OuterMembrane 10.00 | Outside | 0.6353 (Ag) | Non-Allergen | -0.28 | BLAST_ | Information and storage (-1.280) | N/D | 1. Usher: Outer membrane usher protein  2. PapC_N: This N terminal domain is involved in substrate binding and pili assembling  3. PapC_C super family: This domain has a beta-sandwich structure similar to the plug domain of PapC | 100 | 93 | 0.068 | 1.545 |
| 55 | NZ_CP086368_CDS_LMF30_RS12795 | Extracellular 9.71  Secondary localization(s):  Fimbrial | Outside | 0.8350 (Ag) | Non-Allergen | -0.18 | BLAST_ | Virulence factors (1.314) | Fimbrial protein | Type-1 fimbrial protein | 100 | 15 | 0.317 | 1.558 |
| 56 | NZ_CP086368_CDS_bapA | OuterMembrane (This protein may have multiple localization sites.) 10.00 | Outside | 0.7688 (Ag) | Non-Allergen | 0.37 | BLAST_ | Information and storage (-0.915) | outer membrane autotransporter barrel domain | 1. autotrans_barl: outer membrane autotransporter barrel domain [Protein fate, Protein and peptide secretion and trafficking, Cellular processes, Pathogenesis]  2. PRK15313 super family : intestinal colonization autotransporter adhesin MisL | 100 | 91 | 0.344 | 1.665 |
| 57 | NZ_CP086368_CDS_bscC | OuterMembrane 10.00 | Outside | 0.6183 (Ag) | Non-Allergen | -0.49 | BLAST_ | Metabolism Molecule (2.536) | protein transport across the cell outer membrane | EscC/YscC/HrcC family type III secretion system outer membrane ring protein | 100 | 63 | 0.26 | 1.37 |
| 58 | NZ_CP086368_CDS_bscJ | OuterMembrane 9.93  Secondary localization(s):  T3SS | Outside | 0.6362 (Ag) | Non-Allergen | -0.49 | BLAST_ | Cellular process (0.121) | (LipO) protein | type III secretion apparatus lipoprotein, YscJ/HrcJ family [Protein fate, Protein and peptide secretion and trafficking, Cellular processes, Pathogenesis] | 100 | 28 | 0.025 | 1.675 |
| 59 | NZ_CP086368_CDS_LMF30_RS13355 | OuterMembrane 4.74  Extracellular 0.37 | Outside | 0.5728 (Ag) | Non-Allergen | -0.4 | BLAST_ | Virulence factors (1.834) | Outer membrane efflux protein | efflux transporter, outer membrane factor (OMF) lipoprotein, NodT family [Cellular processes, Detoxification, Transport and binding proteins, Porins] | 100 | 35 | 0.06 | 1.19 |
| 60 | NZ_CP086368_CDS_vag8 | OuterMembrane (This protein may have multiple localization sites.) 9.83 | Outside | 0.7973 (Ag) | Non-Allergen | 0.09 | BLAST_ | Virulence factors (-0.297) | outer membrane autotransporter barrel | 1. autotrans_barl: outer membrane autotransporter barrel domain  2. PL1_Passenger_AT: Pertactin-like passenger domains (virulence factors), C-terminal, subgroup 1, of autotransporter proteins of the type V secretion system of Gram-negative bacteria.  3. PRK15319 super family: fibronectin-binding autotransporter adhesin ShdA | 100 | 94 | 0.303 | 1.5 |
| 61 | NZ_CP086368_CDS_LMF30_RS13630 | Extracellular 9.45 | Outside | 0.5118 (Ag) | Non-Allergen | -0.44 | BLAST_ | Cellular process (1.690) | phospholipase C | phospholipase C, phosphocholine-specific, Pseudomonas-type | 100 | 67 | 0.129 | 1.875 |
| 62 | NZ_CP086368_CDS_fauA | OuterMembrane 10.00 | Outside | 0.5225 (Ag) | Non-Allergen | 0.2 | BLAST_ | Cellular process (3.606) | receptor | Outer membrane receptor for ferric coprogen and ferric-rhodotorulic acid [Inorganic ion transport and metabolism] | 100 | 81 | 0.104 | 1.399 |
| 63 | NZ_CP086368_CDS_LMF30_RS14600 | OuterMembrane 9.99 | Outside | 0.5033 (Ag) | Non-Allergen | 0.01 | BLAST_ | Metabolism Molecule (1.148) | Bacterial type II and III secretion system protein | 1. Secretin: Bacterial type II and III secretion system protein  2. CpaC super family: Flp pilus assembly protein, secretin CpaC [Intracellular trafficking, secretion, and vesicular transport, Extracellular structures] | 100 | 49 | 0.208 | 1.489 |
| 64 | NZ_CP086368_CDS_LMF30_RS14800 | Extracellular 9.78 | Outside | 0.6965 (Ag) | Non-Allergen | -0.18 | BLAST_ | Cellular process (0.706) | endonuclease I | Endonuclease I [Replication, recombination and repair] | 100 | 28 | 0.166 | 1.593 |
| 65 | NZ_CP086368_CDS_fimX | Extracellular 10.00  Secondary localization(s):  Fimbrial | Outside | 0.7227 (Ag) | Non-Allergen | 0.03 | BLAST_ | Cellular process (1.438) | Fimbrial protein | Fimbrial protein | 100 | 21 | 0.283 | 1.472 |
| 66 | NZ_CP086368_CDS_LMF30_RS16280 | OuterMembrane 10.00 | Outside | 0.6788 (Ag) | Non-Allergen | -0.12 | BLAST_ | Virulence factors (0.990) | Gram-negative porin | Porins form aqueous channels for the diffusion of small hydrophillic molecules across the outer membrane. | 100 | 39 | 0.2 | 1.563 |
| 67 | NZ_CP086368_CDS_LMF30_RS16730 | OuterMembrane 9.93 | Outside | 0.7061 (Ag) | Non-Allergen | -0.26 | BLAST_ | Virulence factors (2.001) | peptidase | 1. nlpD super family: murein hydrolase activator NlpD  2. LysM: Lysin Motif is a small domain involved in binding peptidoglycan | 99.89 | 23 | 0.215 | 1.629 |
| 68 | NZ_CP086368_CDS_LMF30_RS16745 | OuterMembrane 10.00 | Outside | 0.6063 (Ag) | Non-Allergen | -0.34 | BLAST_ | Cellular process (0.481) | TonB-dependent receptor | TonB dependent/Ligand-Gated channels are created by a monomeric 22 strand (22,24) anti-parallel beta-barrel. | 99.89 | 79 | 0.144 | 1.404 |
| 69 | NZ_CP086368_CDS_LMF30_RS16915 | OuterMembrane 9.93 | Outside | 0.5011 (Ag) | Non-Allergen | 0.06 | BLAST_ | Virulence factors (1.773) | peptidase | putative peptidase; Provisional | 100 | 51 | 0.129 | 1.305 |
| 70 | NZ_CP086368_CDS_LMF30_RS17210 | OuterMembrane 9.93 | Outside | 0.5254 (Ag) | Non-Allergen | -0.44 | BLAST_ | Cellular process (1.998) | peptidase | Murein DD-endopeptidase MepM and murein hydrolase activator NlpD, contain LysM domain [Cell wall/membrane/envelope biogenesis] | 100 | 34 | 0.051 | 1.554 |
| 71 | NZ_CP086368_CDS_LMF30_RS17550 | OuterMembrane 10.00 | Outside | 0.7347 (Ag) | Non-Allergen | 0.06 | BLAST_ | Metabolism Molecule (0.469) | TonB-dependent receptor | zinc piracy TonB-dependent receptor ZnuD | 100 | 77 | 0.155 | 1.586 |
| 72 | NZ_CP086368_CDS_LMF30_RS18280 | OuterMembrane 9.49 | Outside | 0.5567 (Ag) | Non-Allergen | 0.12 | BLAST_ | Cellular process (2.688) | N/D | 1. FimD super family: Outer membrane usher protein FimD/PapC [Cell motility, Extracellular structures]  2. PRK07003 super family: DNA polymerase III subunit gamma/tau | 100 | 43 | 0.147 | 1.838 |
| 73 | NZ_CP086368_CDS_LMF30_RS18775 | OuterMembrane 9.93 | Outside | 0.7096 (Ag) | Non-Allergen | 0.13 | BLAST_ | Cellular process (1.453) | copper resistance | Copper resistance protein B precursor (CopB) | 99.23 | 31 | 0.128 | 1.763 |
| 74 | NZ_CP086368_CDS_LMF30_RS18870 | OuterMembrane 10.00 | Outside | 0.6507 (Ag) | Non-Allergen | 0.03 | BLAST_ | Virulence factors (1.515) | Receptor | Outer membrane receptor for monomeric catechols [Inorganic ion transport and metabolism] | 7.86 | 63 | 0.11 | 1.415 |
| 75 | NZ_CP086368_CDS_bopC | OuterMembrane 9.49 | Outside | 0.7764 (Ag) | Non-Allergen | 0.02 | BLAST_ | Virulence factors (-0.917) | Not Available | 1. DUF3220: Protein of unknown function (DUF3120); This family of proteins with unknown function appears to be restricted to Bordetella.  2. toxin_BteA-MLD_like: membrane localization domain (MLD) of BteA (Bordetella T3SS effector A) cytotoxin, the N-terminal domain of Photox toxin and related proteins | 100 | 68 | 0.06 | 1.448 |
| 76 | NZ_CP086368_CDS_fliF | Cytoplasmic Membrane 10.00  Secondary localization(s):  Flagellar | Outside | 0.5806 (Ag) | Non-Allergen | 0.09 | BLAST_ | Metabolism Molecule (1.721) | bacterial-type flagellum-dependent cell motility | COG1766, Flagellar biosynthesis/type III secretory pathway M-ring protein FliF/YscJ [Cell motility, Intracellular trafficking, secretion, and vesicular transport] | 100 | 61 | 0.179 | 1.489 |
