# Supplementary material for: Exploiting subtractive genomics to identify novel drug targets and new immunogenic candidates against Bordetella pertussis: an in silico study
Source: Front Bioinform. 2025 May 13;5:1570054. doi: 10.3389/fbinf.2025.1570054 (PMC12106433; doi:10.3389/fbinf.2025.1570054)
Supplement: Supplementary file 4 [file DataSheet3.docx]

**Table S3**. PDB availability of 59 cytoplasmic proteins that were assessed to identify novel drug targets against *B. pertussis*.

| **No.** | **Protein** | **Accession number** | **Protein target** | **PDB target** | **Coverage** | **Identity** |
| --- | --- | --- | --- | --- | --- | --- |
| 1 | ParB family protein | WP_010929571.1 | Chain A, ParB family protein [*Myxococcus xanthus* DK 1622] | 7BNK_A | 66% | 51.67% |
| 2 | UMPkinase | WP_010930349.1 | Chain A, Uridylate kinase [*Neisseria meningitidis*] | 1YBD_A | 97% | 66.52% |
| 3 | orotidine-5'-phosphate decarboxylase | WP_003808606.1 | Chain A, Orotidine-5-phosphate decarboxylase/orotate phosphoribosyltransferase, putative (Ompdcase-oprtase, putative) [*Leishmania infantum*] | 3QW3_A | 89% | 45.90% |
| 4 | imidazole glycerol phosphate synthase subunit HisF | WP_003815802.1 | Chain A, HisF-LUCA [synthetic construct] | 4EVZ_A | 94% | 67,84% |
| 5 | ScpA family protein | WP_003815053.1 | Chain A, ScpA [*Geobacillus stearothermophilus*] | 3W6J_A | 34% | 47.32% |
| 6 | N-acetyl-gamma-glutamyl-phosphate reductase | WP_003820599.1 | Chain A, putative N-acetyl-gamma-glutamyl-phosphate reductase [*Arabidopsis thaliana*] | 1XYG_A | 97% | 43.27% |
| 7 | DUF1841 family protein | WP_003814061.1 | No significant similarity found. | | | |
| 8 | 50S ribosomal protein L35 | WP_003812834.1 | Chain 2, 50S ribosomal protein L35 [*Acinetobacter baumannii* AB0057] | 6V39_2 | 98% | 59.38% |
| 9 | elongation factor P | WP_003810194.1 | Chain A, Elongation factor P [*Pseudomonas aeruginosa*] | 3OYY_A | 99% | 63.44% |
| 10 | pyruvate, water dikinase regulatory protein | WP_010930357.1 | Chain A, Pyruvate, phosphate dikinase regulatory protein, chloroplastic | 5D0N_A | 94% | 34.27% |
| 11 | 50S ribosomal protein L35 | WP_003812834.1 | Chain 2, 50S ribosomal protein L35 [*Acinetobacter baumannii* AB0057] | 6V39_2 | 98% | 59.38% |
| 12 | imidazoleglycerol-phosphate dehydratase HisB | WP_003815795.1 | Chain A, Imidazoleglycerol-phosphate dehydratase 1 [*Arabidopsis thaliana*] | 2F1D_A | 99% | 52.58% |
| 13 | 50S ribosomal protein L29 | WP_003806912.1 | Chain A, 50S ribosomal protein L29 [*Pseudomonas aeruginosa* PAO1] | 7UNR_1 | 100% | 55.56% |
| 14 | HPr(Ser) kinase/phosphatase | WP_010929965.1 | Chain A, Hpr kinase/phosphatase [*Staphylococcus xylosus*] | 1KO7_A | 99% | 37.79% |
| 15 | 50S ribosomal protein L18 | WP_003806922.1 | Select seq pdb\|8RD8\|Nc Chain Nc, Large ribosomal subunit protein uL18 [*Psychrobacter urativorans*] | 8RD8_Nc | 100% | 60.83% |
| 16 | 30S ribosomal protein S21 | WP_006218592.1 | Chain v, 30S ribosomal protein S21 [*Escherichia coli* K-12] | 7QG8_v | 92% | 63.08% |
| 17 | flagellar motor switch protein FliN | WP_010930335.1 | Chain AB, Flagellar motor switch protein FliN [*Salmonella enterica* subsp. enterica serovar Typhimurium] | 8T8O_AB | 71% | 68.33% |
| 18 | primosomal protein N' | WP_019247688.1 | Chain H, Primosome assembly protein PriA [*Klebsiella pneumoniae* MGH 78578] | 4NL4_H | 96% | 40.22% |
| 19 | 30S ribosomal protein S6 | WP_010926331.1 | Chain f, 30S ribosomal protein S6 [*Acinetobacter baumannii* AB0057] | 6V39_f | 94% | 60.50% |
| 20 | SsrA-binding protein SmpB | WP_003811754.1 | Chain 5, SsrA-binding protein [*Escherichia coli* K-12] | 7ACJ_5 | 95% | 45.95% |
| 21 | protein phosphatase CheZ | WP_003817162.1 | Chain Z, Chemotaxis protein cheZ [*Escherichia coli*] | 1KMI_Z | 93% | 65.50% |
| 22 | 50S ribosomal protein L24 | WP_003806917.1 | Chain U, 50S ribosomal protein L24 [*Escherichia coli* K-12] | 3J7Z_U | 93% | 58.59% |
| 23 | 50S ribosomal protein L33 | WP_010930711.1 | Chain 1, 50S ribosomal protein L33 [*Escherichia coli* K-12] | 3J7Z_1 | 100% | 76.36% |
| 24 | ketol-acid reductoisomerase | WP_010930015.1 | Chain A, Ketol-acid reductoisomerase [*Pseudomonas aeruginosa*] | 1NP3_A | 100% | 71.01% |
| 25 | type B 50S ribosomal protein L31 | WP_003818624.1 | Chain a, 50S ribosomal protein L31 [*Flavobacterium johnsoniae*] | 7JIL_a | 92% | 47.56% |
| 26 | integration host factor subunit alpha | WP_010931006.1 | Chain A, PROTEIN (INTEGRATION HOST FACTOR (ALPHA) (IHF)) [*Escherichia coli*] | 1IHF_A | 88% | 68.04% |
| 27 | aspartate kinase | WP_010930633.1 | Chain A, Aspartokinase [*Pseudomonas aeruginosa* PAO1] | 5YEI_A | 98% | 63.13% |
| 28 | transcription termination/antitermination protein NusG | WP_003806885.1 | Chain A, Transcription antitermination protein nusG [*Escherichia coli*] | 2JVV_A | 98% | 60.00% |
| 29 | ATP-dependent protease subunit HslV | WP_010931291.1 | Chain A, ATP-dependent protease subunit HslV [*Escherichia coli* 55989] | 5JI2_A | 95% | 67.25% |
| 30 | 50S ribosomal protein L36 | WP_003806928.1 | Chain 6, 50S ribosomal protein L36 [*Pseudomonas aeruginosa*] | 6SPB_6 | 100% | 73.68% |
| 31 | transcriptional repressor LexA | WP_010930566.1 | Chain A, LexA repressor [*Escherichia coli* K-12] | 7B5G_A | 96% | 58.85% |
| 32 | sulfurtransferase TusA family protein | WP_004568544.1 | Chain A, Hypothetical protein Ta1170/Ta1414 [*Thermoplasma acidophilum*] | 1PAV_A | 80% | 29.41% |
| 33 | 30S ribosome-binding factor RbfA | WP_003818636.1 | Chain V, Ribosome-binding factor A [*Escherichia coli*] | 7AFL_V | 87% | 37.50% |
| 34 | 1-(5-phosphoribosyl)-5-[(5-phosphoribosylamino)methylideneamino]imidazole-4-carboxamide isomerase | WP_010927224.1 | Chain A, 1-(5-phosphoribosyl)-5-[(5-phosphoribosylamino)methylideneamino] imidazole-4-carboxamide isomerase [*Paenarthrobacter aurescens*] | 4WD0_A | 95% | 37.45% |
| 35 | 50S ribosomal protein L6 | WP_010931568.1 | Chain G, 50S ribosomal protein L6 [*Pseudomonas aeruginosa* PAO1] | 7UNR_G | 100% | 59.32% |
| 36 | 50S ribosomal protein L21 | WP_003807462.1 | Chain Q, 50S ribosomal protein L21 [*Acinetobacter baumannii* AB0057] | 6V39_Q | 100% | 59.22% |
| 37 | sulfate adenylyltransferase subunit CysD | WP_224019510.1 | Chain A, Sulfate adenylyltransferase subunit 2 [*Pseudomonas syringae*] | 1ZUN_A | 97% | 43.71% |
| 38 | 50S ribosomal protein L23 | WP_003806906.1 | Chain V, 50S ribosomal protein L23 [*Pseudomonas aeruginosa* PAO1] | 7UNR_V | 96% | 52.63% |
| 39 | chromosomal replication initiator protein DnaA | WP_010929554.1 | Chain A, Chromosomal replication initiator protein DnaA [*Bacillus subtilis*] | 8BTG_A | 99% | 38.33% |
| 40 | GTP cyclohydrolase FolE2 | WP_003813103.1 | Chain A, GTP cyclohydrolase FolE2 [*Burkholderia pseudomallei*] | 8G6C_A | 98% | 61.15% |
| 41 | phosphoribosyl-AMP cyclohydrolase | WP_010931645.1 | Chain A, Phosphoribosyl-AMP cyclohydrolase [*Methanothermobacter thermautotrophicus*] | 1ZPS_A | 86% | 55.17% |
| 42 | homoserine dehydrogenase | WP_003813074.1 | Chain A, Homoserine dehydrogenase [*Thiobacillus denitrificans* ATCC 25259] | 3MTJ_A | 94% | 69.90% |
| 43 | translation initiation factor IF-1 | WP_003806927.1 | Chain A, Translation initiation factor IF-1 [*Burkholderia thailandensis* E264] | 2N3S_A | 100% | 94.44% |
| 44 | acetolactate synthase small subunit | WP_003814006.1 | Chain A, Probable acetolactate synthase isozyme III (Small subunit) [*Nitrosomonas europaea* ATCC 19718] | 2PC6_A | 99% | 66.67% |
| 45 | 50S ribosomal protein L28 | WP_003810297.1 | Chain Z, 50S ribosomal protein L28 [*Pseudomonas aeruginosa* PAO1] | 7UNR_Z | 97% | 78.95% |
| 46 | Dihydroxy-acid dehydratase | WP_003819105.1 | Chain A, Dihydroxy-acid dehydratase [*Synechocystis* sp. PCC 6803] | 6NTE_A | 98% | 38.93% |
| 47 | cell division topological specificity factor MinE | WP_003814252.1 | Chain A, Cell division topological specificity factor [*Neisseria gonorrhoeae*] | 2KXO_A | 98% | 45.78% |
| 48 | Chemotaxis protein CheW | WP_003811919.1 | Chain A, Chemotaxis protein cheW [*Escherichia coli*] | 2HO9_A | 100% | 70.48% |
| 49 | Preprotein translocase subunit SecA | WP_003814564.1 | Chain A, Preprotein translocase secA subunit [*Escherichia coli*] | 2FSF_A | 92% | 60.17% |
| 50 | carboxynorspermidine decarboxylase | WP_003814461.1 | Chain A, Putative carboxynorspermidine decarboxylase protein [*Sinorhizobium meliloti*] | 3MT1_A | 99% | 67.03% |
| 51 | flavodoxin-dependent (E)-4-hydroxy-3-methylbut-2-enyl-diphosphate synthase | WP_010930793.1 | Chain A, 4-hydroxy-3-methylbut-2-en-1-yl diphosphate synthase [*Thermus thermophilus* HB8] | 4S38_A | 93% | 60.20% |
| 52 | RNA polymerase-binding protein DksA | WP_003807164.1 | Chain A, Putative C4-type zinc finger protein, DksA/TraR family [*Pseudomonas aeruginosa* UCBPP-PA14] | 4IJJ_A | 83% | 47.66% |
| 53 | PTS sugar transporter subunit IIA | WP_010929966.1 | Chain A, PTS IIA-like nitrogen-regulatory protein PtsN [*Burkholderia pseudomallei* 1710b] | 4GQX_A | 99% | 65.33% |
| 54 | 50S ribosomal protein L32 | WP_003813827.1 | Chain 4, 50S ribosomal protein L32 [*Pseudomonas aeruginosa* PAO1] | 7UNR_4 | 100% | 65.00% |
| 55 | redox-sensitive transcriptional activator SoxR | WP_003820813.1 | Chain A, Redox-sensitive transcriptional activator soxR [*Escherichia coli* K-12] | 2ZHG_A | 92% | 57.75% |
| 56 | glutamyl-tRNA reductase | WP_010929957.1 | Chain A, Glutamyl-tRNA reductase 1, chloroplastic [*Arabidopsis thaliana*] | 4N7R_A | 95% | 31.19% |
| 57 | endonuclease/exonuclease/phosphatase family protein | WP_003811372.1 | No significant similarity found. | | | |
| 58 | 50S ribosomal protein L19 | WP_003813315.1 | Chain A, Translation initiation factor IF-3 [*Escherichia coli*] | 8JSG_A | 94% | 69.94% |
| 59 | translation initiation factor IF-3 | WP_033446215.1_1483 | Chain O, 50S ribosomal protein L19 [*Acinetobacter baumannii* AB0057] | 6V39_O | 81% | 74.76% |
