# Supplementary material for: Exploiting subtractive genomics to identify novel drug targets and new immunogenic candidates against Bordetella pertussis: an in silico study
Source: Front Bioinform. 2025 May 13;5:1570054. doi: 10.3389/fbinf.2025.1570054 (PMC12106433; doi:10.3389/fbinf.2025.1570054)
Supplement: Supplementary file 8 [file DataSheet1.docx]

**Table S1.** The list of 554 *B. pertussis* genomes has been used in this study.

1. Bordetella_pertussis_137_ NZ_CP010323
2. Bordetella_pertussis_18323_ NC_018518
3. Bordetella_pertussis_B1917_ CP009751
4. Bordetella_pertussis_B1920_ CP009752
5. Bordetella_pertussis_CS_ NC_017223
6. Bordetella_pertussis_Tohama_I_ NC_002929
7. Bordetella_pertussis_Tohama_I_ NZ_CP039021
8. Bordetella_pertussis_Tohama_I_ NZ_CP039022
9. Bordetella_pertussis_chromosome_I_ NZ_LR130529
10. Bordetella_pertussis_strain_A339_ NZ_CP026634
11. Bordetella_pertussis_strain_B1838_ NZ_CP011440
12. Bordetella_pertussis_strain_B1865_ NZ_CP011441
13. Bordetella_pertussis_strain_B199_ NZ_CP022361
14. Bordetella_pertussis_strain_B201_ NZ_CP013075
15. Bordetella_pertussis_strain_B202_ NZ_CP016338
16. Bordetella_pertussis_strain_B203_ NZ_CP012128
17. Bordetella_pertussis_strain_B226_ NZ_CP016957
18. Bordetella_pertussis_strain_B227_ NZ_CP013076
19. Bordetella_pertussis_strain_B228_ NZ_CP026465
20. Bordetella_pertussis_strain_B3405_ NZ_CP011442
21. Bordetella_pertussis_strain_B3582_ NZ_CP011443
22. Bordetella_pertussis_strain_B3585_ NZ_CP011444
23. Bordetella_pertussis_strain_B3621_ NZ_CP011401
24. Bordetella_pertussis_strain_B3629_ NZ_CP011400
25. Bordetella_pertussis_strain_B3640_ NZ_CP011445
26. Bordetella_pertussis_strain_B3658_ NZ_CP011446
27. Bordetella_pertussis_strain_B3913_ NZ_CP011447
28. Bordetella_pertussis_strain_B3921_ NZ_CP011448
29. Bordetella_pertussis_strain_BPD1_ NZ_CP034182
30. Bordetella_pertussis_strain_BPD2_ NZ_CP034101
31. Bordetella_pertussis_strain_C393_ NZ_CP010963
32. Bordetella_pertussis_strain_C505_ NZ_CP011687
33. Bordetella_pertussis_strain_C549_ NZ_CP013077
34. Bordetella_pertussis_strain_C569_ NZ_CP025347
35. Bordetella_pertussis_strain_C571_ NZ_CP011167
36. Bordetella_pertussis_strain_C734_ NZ_CP013078
37. Bordetella_pertussis_strain_C742_ NZ_CP011688
38. Bordetella_pertussis_strain_C756_ NZ_CP025368
39. Bordetella_pertussis_strain_C757_ NZ_CP013079
40. Bordetella_pertussis_strain_C871_ NZ_CP025345
41. Bordetella_pertussis_strain_C927_ NZ_CP016339
42. Bordetella_pertussis_strain_C934_ NZ_CP016961
43. Bordetella_pertussis_strain_C958_ NZ_CP011168
44. Bordetella_pertussis_strain_C975_ NZ_CP013868
45. Bordetella_pertussis_strain_D175_ NZ_CP011689
46. Bordetella_pertussis_strain_D236_ NZ_CP025530
47. Bordetella_pertussis_strain_D321_ NZ_CP011690
48. Bordetella_pertussis_strain_D322_ NZ_CP025358
49. Bordetella_pertussis_strain_D420_chromosome_1_ NZ_LN849008
50. Bordetella_pertussis_strain_D422_ NZ_CP016959
51. Bordetella_pertussis_strain_D502_ NZ_CP011691
52. Bordetella_pertussis_strain_D521_ NZ_CP011169
53. Bordetella_pertussis_strain_D665_ NZ_CP025526
54. Bordetella_pertussis_strain_D717_ NZ_CP016964
55. Bordetella_pertussis_strain_D735_ NZ_CP016960
56. Bordetella_pertussis_strain_D799_ NZ_CP016963
57. Bordetella_pertussis_strain_D869_ NZ_CP025528
58. Bordetella_pertussis_strain_D879_ NZ_CP011170
59. Bordetella_pertussis_strain_D919_ NZ_CP025355
60. Bordetella_pertussis_strain_D925_ NZ_CP016968
61. Bordetella_pertussis_strain_E024_ NZ_CP011692
62. Bordetella_pertussis_strain_E025_ NZ_CP016967
63. Bordetella_pertussis_strain_E087_ NZ_CP025480
64. Bordetella_pertussis_strain_E140_ NZ_CP025354
65. Bordetella_pertussis_strain_E150_ NZ_CP011171
66. Bordetella_pertussis_strain_E153_ NZ_CP025359
67. Bordetella_pertussis_strain_E191_ NZ_CP025478
68. Bordetella_pertussis_strain_E194_ NZ_CP013080
69. Bordetella_pertussis_strain_E198_ NZ_CP025385
70. Bordetella_pertussis_strain_E365_ NZ_CP025387
71. Bordetella_pertussis_strain_E368_ NZ_CP013869
72. Bordetella_pertussis_strain_E476_ NZ_CP010964
73. Bordetella_pertussis_strain_E530_ NZ_CP011693
74. Bordetella_pertussis_strain_E537_ NZ_CP016958
75. Bordetella_pertussis_strain_E541_ NZ_CP016966
76. Bordetella_pertussis_strain_E555_ NZ_CP011172
77. Bordetella_pertussis_strain_E587_ NZ_CP011173
78. Bordetella_pertussis_strain_E602_ NZ_CP013081
79. Bordetella_pertussis_strain_E809_ NZ_CP011174
80. Bordetella_pertussis_strain_E898_ NZ_CP016962
81. Bordetella_pertussis_strain_E945_ NZ_CP016956
82. Bordetella_pertussis_strain_E976_ NZ_CP011175
83. Bordetella_pertussis_strain_F011_ NZ_CP011176
84. Bordetella_pertussis_strain_F013_ NZ_CP016965
85. Bordetella_pertussis_strain_F034_ NZ_CP011177
86. Bordetella_pertussis_strain_F501_ NZ_CP013870
87. Bordetella_pertussis_strain_F569_ NZ_CP025523
88. Bordetella_pertussis_strain_F578_ NZ_CP025357
89. Bordetella_pertussis_strain_F580_ NZ_CP025342
90. Bordetella_pertussis_strain_F657_ NZ_CP013871
91. Bordetella_pertussis_strain_F658_ NZ_CP011178
92. Bordetella_pertussis_strain_F670_ NZ_CP011179
93. Bordetella_pertussis_strain_F684_ NZ_CP011180
94. Bordetella_pertussis_strain_F687_ NZ_CP011181
95. Bordetella_pertussis_strain_F778_ NZ_CP013872
96. Bordetella_pertussis_strain_F934_ NZ_CP013873
97. Bordetella_pertussis_strain_F948_ NZ_CP011182
98. Bordetella_pertussis_strain_F954_ NZ_CP025366
99. Bordetella_pertussis_strain_FDAARGOS_179_ CP014153
100. Bordetella_pertussis_strain_G057_ NZ_CP012129
101. Bordetella_pertussis_strain_G085_ NZ_CP013874
102. Bordetella_pertussis_strain_G102_ NZ_CP025388
103. Bordetella_pertussis_strain_G807_ NZ_CP013875
104. Bordetella_pertussis_strain_G965_ NZ_CP013876
105. Bordetella_pertussis_strain_H034_ NZ_CP025356
106. Bordetella_pertussis_strain_H320_ NZ_CP011234
107. Bordetella_pertussis_strain_H321_ NZ_CP010965
108. Bordetella_pertussis_strain_H346_ NZ_CP011694
109. Bordetella_pertussis_strain_H348_ NZ_CP013877
110. Bordetella_pertussis_strain_H361_ NZ_CP013878
111. Bordetella_pertussis_strain_H374_ NZ_CP010838
112. Bordetella_pertussis_strain_H375_ NZ_CP010961
113. Bordetella_pertussis_strain_H378_ NZ_CP010839
114. Bordetella_pertussis_strain_H379_ NZ_CP010840
115. Bordetella_pertussis_strain_H380_ NZ_CP010841
116. Bordetella_pertussis_strain_H382_ NZ_CP013082
117. Bordetella_pertussis_strain_H437_ NZ_CP011695
118. Bordetella_pertussis_strain_H489_ NZ_CP010842
119. Bordetella_pertussis_strain_H520_ NZ_CP011183
120. Bordetella_pertussis_strain_H533_ NZ_CP013879
121. Bordetella_pertussis_strain_H540_ NZ_CP013880
122. Bordetella_pertussis_strain_H541_ NZ_CP025373
123. Bordetella_pertussis_strain_H542_ NZ_CP010843
124. Bordetella_pertussis_strain_H559_ NZ_CP010844
125. Bordetella_pertussis_strain_H561_ NZ_CP010845
126. Bordetella_pertussis_strain_H563_ NZ_CP010846
127. Bordetella_pertussis_strain_H564_ NZ_CP010249
128. Bordetella_pertussis_strain_H579_ NZ_CP011184
129. Bordetella_pertussis_strain_H622_ NZ_CP010847
130. Bordetella_pertussis_strain_H624_ NZ_CP025529
131. Bordetella_pertussis_strain_H627_ NZ_CP010962
132. Bordetella_pertussis_strain_H636_ NZ_CP013881
133. Bordetella_pertussis_strain_H637_ NZ_CP011185
134. Bordetella_pertussis_strain_H639_ NZ_CP012130
135. Bordetella_pertussis_strain_H640_ NZ_CP025371
136. Bordetella_pertussis_strain_H642_ NZ_CP025360
137. Bordetella_pertussis_strain_H665_ NZ_CP011186
138. Bordetella_pertussis_strain_H672_ NZ_CP025349
139. Bordetella_pertussis_strain_H677_ NZ_CP025367
140. Bordetella_pertussis_strain_H681_ NZ_CP012078
141. Bordetella_pertussis_strain_H682_ NZ_CP013083
142. Bordetella_pertussis_strain_H696_ NZ_CP021402
143. Bordetella_pertussis_strain_H697_ NZ_CP025365
144. Bordetella_pertussis_strain_H698_ NZ_CP013084
145. Bordetella_pertussis_strain_H703_ NZ_CP011187
146. Bordetella_pertussis_strain_H706_ NZ_CP013085
147. Bordetella_pertussis_strain_H707_ NZ_CP011188
148. Bordetella_pertussis_strain_H709_ NZ_CP025364
149. Bordetella_pertussis_strain_H710_ NZ_CP011236
150. Bordetella_pertussis_strain_H729_ NZ_CP011189
151. Bordetella_pertussis_strain_H730_ NZ_CP013086
152. Bordetella_pertussis_strain_H740_ NZ_CP011190
153. Bordetella_pertussis_strain_H742_ NZ_CP025346
154. Bordetella_pertussis_strain_H754_ NZ_CP011191
155. Bordetella_pertussis_strain_H762_ NZ_CP011696
156. Bordetella_pertussis_strain_H763_ NZ_CP011697
157. Bordetella_pertussis_strain_H764_ NZ_CP011698
158. Bordetella_pertussis_strain_H765_ NZ_CP011192
159. Bordetella_pertussis_strain_H766_ NZ_CP011699
160. Bordetella_pertussis_strain_H768_ NZ_CP011700
161. Bordetella_pertussis_strain_H771_ NZ_CP013087
162. Bordetella_pertussis_strain_H773_ NZ_CP011701
163. Bordetella_pertussis_strain_H775_ NZ_CP011702
164. Bordetella_pertussis_strain_H778_ NZ_CP025362
165. Bordetella_pertussis_strain_H779_ NZ_CP011703
166. Bordetella_pertussis_strain_H782_ NZ_CP018035
167. Bordetella_pertussis_strain_H784_ NZ_CP011193
168. Bordetella_pertussis_strain_H787_ NZ_CP011704
169. Bordetella_pertussis_strain_H788_ NZ_CP010250
170. Bordetella_pertussis_strain_H800_ NZ_CP011194
171. Bordetella_pertussis_strain_H806_ NZ_CP011195
172. Bordetella_pertussis_strain_H810_ NZ_CP011196
173. Bordetella_pertussis_strain_H811_ NZ_CP025361
174. Bordetella_pertussis_strain_H812_ NZ_CP011197
175. Bordetella_pertussis_strain_H813_ NZ_CP025351
176. Bordetella_pertussis_strain_H814_ NZ_CP025374
177. Bordetella_pertussis_strain_H834_ NZ_CP011235
178. Bordetella_pertussis_strain_H842_ NZ_CP011705
179. Bordetella_pertussis_strain_H847_ NZ_CP011706
180. Bordetella_pertussis_strain_H849_ NZ_CP011707
181. Bordetella_pertussis_strain_H851_ NZ_CP011237
182. Bordetella_pertussis_strain_H852_ NZ_CP012079
183. Bordetella_pertussis_strain_H853_ NZ_CP011708
184. Bordetella_pertussis_strain_H864_ NZ_CP011709
185. Bordetella_pertussis_strain_H866_ NZ_CP011710
186. Bordetella_pertussis_strain_H876_ NZ_CP013882
187. Bordetella_pertussis_strain_H877_ NZ_CP025382
188. Bordetella_pertussis_strain_H878_ NZ_CP011711
189. Bordetella_pertussis_strain_H883_ NZ_CP011712
190. Bordetella_pertussis_strain_H884_ NZ_CP011713
191. Bordetella_pertussis_strain_H902_ NZ_CP025363
192. Bordetella_pertussis_strain_H910_ NZ_CP011714
193. Bordetella_pertussis_strain_H911_ NZ_CP011238
194. Bordetella_pertussis_strain_H915_ NZ_CP011239
195. Bordetella_pertussis_strain_H920_ NZ_CP025352
196. Bordetella_pertussis_strain_I069_ NZ_CP011715
197. Bordetella_pertussis_strain_I075_ NZ_CP011240
198. Bordetella_pertussis_strain_I088_ NZ_CP011716
199. Bordetella_pertussis_strain_I089_ NZ_CP011717
200. Bordetella_pertussis_strain_I093_ NZ_CP013883
201. Bordetella_pertussis_strain_I094_ NZ_CP018036
202. Bordetella_pertussis_strain_I106_ NZ_CP012080
203. Bordetella_pertussis_strain_I110_ NZ_CP011718
204. Bordetella_pertussis_strain_I111_ NZ_CP011719
205. Bordetella_pertussis_strain_I112_ NZ_CP011241
206. Bordetella_pertussis_strain_I113_ NZ_CP011720
207. Bordetella_pertussis_strain_I120_ NZ_CP025370
208. Bordetella_pertussis_strain_I127_ NZ_CP011721
209. Bordetella_pertussis_strain_I135_ NZ_CP011722
210. Bordetella_pertussis_strain_I136_ NZ_CP011723
211. Bordetella_pertussis_strain_I150_ NZ_CP012131
212. Bordetella_pertussis_strain_I182_ NZ_CP026996
213. Bordetella_pertussis_strain_I187_ NZ_CP012132
214. Bordetella_pertussis_strain_I188_ NZ_CP025379
215. Bordetella_pertussis_strain_I223_ NZ_CP025369
216. Bordetella_pertussis_strain_I228_ NZ_CP011198
217. Bordetella_pertussis_strain_I238_ NZ_CP011199
218. Bordetella_pertussis_strain_I257_ NZ_CP011724
219. Bordetella_pertussis_strain_I259_ NZ_CP012133
220. Bordetella_pertussis_strain_I263_ NZ_CP011725
221. Bordetella_pertussis_strain_I270_ NZ_CP011726
222. Bordetella_pertussis_strain_I271_ NZ_CP011727
223. Bordetella_pertussis_strain_I273_ NZ_CP011728
224. Bordetella_pertussis_strain_I289_ NZ_CP011729
225. Bordetella_pertussis_strain_I315_ NZ_CP011730
226. Bordetella_pertussis_strain_I318_ NZ_CP011731
227. Bordetella_pertussis_strain_I323_ NZ_CP025377
228. Bordetella_pertussis_strain_I331_ NZ_CP011732
229. Bordetella_pertussis_strain_I344_ NZ_CP011255
230. Bordetella_pertussis_strain_I350_ NZ_CP011733
231. Bordetella_pertussis_strain_I351_ NZ_CP012081
232. Bordetella_pertussis_strain_I372_ NZ_CP025372
233. Bordetella_pertussis_strain_I373_ NZ_CP011200
234. Bordetella_pertussis_strain_I375_ NZ_CP011734
235. Bordetella_pertussis_strain_I379_ NZ_CP011735
236. Bordetella_pertussis_strain_I380_ NZ_CP011736
237. Bordetella_pertussis_strain_I382_ NZ_CP011737
238. Bordetella_pertussis_strain_I384_ NZ_CP017882
239. Bordetella_pertussis_strain_I385_ NZ_CP017883
240. Bordetella_pertussis_strain_I386_ NZ_CP011738
241. Bordetella_pertussis_strain_I387_ NZ_CP011201
242. Bordetella_pertussis_strain_I420_ NZ_CP025525
243. Bordetella_pertussis_strain_I439_ NZ_CP025375
244. Bordetella_pertussis_strain_I452_ NZ_CP011739
245. Bordetella_pertussis_strain_I461_ NZ_CP011740
246. Bordetella_pertussis_strain_I462_ NZ_CP025376
247. Bordetella_pertussis_strain_I464_ NZ_CP012134
248. Bordetella_pertussis_strain_I468_ NZ_CP010251
249. Bordetella_pertussis_strain_I469_ NZ_CP010252
250. Bordetella_pertussis_strain_I472_ NZ_CP010253
251. Bordetella_pertussis_strain_I475_ NZ_CP010347
252. Bordetella_pertussis_strain_I476_ NZ_CP010254
253. Bordetella_pertussis_strain_I480_ NZ_CP010255
254. Bordetella_pertussis_strain_I483_ NZ_CP010256
255. Bordetella_pertussis_strain_I496_ NZ_CP010257
256. Bordetella_pertussis_strain_I498_ NZ_CP010258
257. Bordetella_pertussis_strain_I518_ NZ_CP010259
258. Bordetella_pertussis_strain_I521_ NZ_CP010260
259. Bordetella_pertussis_strain_I538_ NZ_CP010261
260. Bordetella_pertussis_strain_I539_ NZ_CP010262
261. Bordetella_pertussis_strain_I598_ NZ_CP025380
262. Bordetella_pertussis_strain_I602_ NZ_CP011202
263. Bordetella_pertussis_strain_I623_ NZ_CP025386
264. Bordetella_pertussis_strain_I646_ NZ_CP010263
265. Bordetella_pertussis_strain_I656_ NZ_CP010264
266. Bordetella_pertussis_strain_I669_ NZ_CP010265
267. Bordetella_pertussis_strain_I692_ NZ_CP025378
268. Bordetella_pertussis_strain_I705_ NZ_CP025524
269. Bordetella_pertussis_strain_I707_ NZ_CP010266
270. Bordetella_pertussis_strain_I728_ NZ_CP011741
271. Bordetella_pertussis_strain_I730_ NZ_CP011203
272. Bordetella_pertussis_strain_I735_ NZ_CP011742
273. Bordetella_pertussis_strain_I743_ NZ_CP012082
274. Bordetella_pertussis_strain_I751_ NZ_CP012083
275. Bordetella_pertussis_strain_I752_ NZ_CP011204
276. Bordetella_pertussis_strain_I754_ NZ_CP011743
277. Bordetella_pertussis_strain_I755_ NZ_CP011744
278. Bordetella_pertussis_strain_I762_ NZ_CP011745
279. Bordetella_pertussis_strain_I763_ NZ_CP011205
280. Bordetella_pertussis_strain_I859_ NZ_CP025477
281. Bordetella_pertussis_strain_I892_ NZ_CP025479
282. Bordetella_pertussis_strain_I896_ NZ_CP025381
283. Bordetella_pertussis_strain_I915_ NZ_CP011206
284. Bordetella_pertussis_strain_I944_ NZ_CP011207
285. Bordetella_pertussis_strain_I945_ NZ_CP025384
286. Bordetella_pertussis_strain_I955_ NZ_CP025531
287. Bordetella_pertussis_strain_I958_ NZ_CP025350
288. Bordetella_pertussis_strain_I959_ NZ_CP011746
289. Bordetella_pertussis_strain_I965_ NZ_CP011747
290. Bordetella_pertussis_strain_I968_ NZ_CP011748
291. Bordetella_pertussis_strain_I975_ NZ_CP011242
292. Bordetella_pertussis_strain_I976_ NZ_CP012084
293. Bordetella_pertussis_strain_I977_ NZ_CP011749
294. Bordetella_pertussis_strain_I978_ NZ_CP011750
295. Bordetella_pertussis_strain_I979_ NZ_CP010966
296. Bordetella_pertussis_strain_I998_ NZ_CP011243
297. Bordetella_pertussis_strain_J010_ NZ_CP012085
298. Bordetella_pertussis_strain_J012_ NZ_CP011751
299. Bordetella_pertussis_strain_J013_ NZ_CP012086
300. Bordetella_pertussis_strain_J014_ NZ_CP012135
301. Bordetella_pertussis_strain_J016_ NZ_CP011752
302. Bordetella_pertussis_strain_J018_ NZ_CP011208
303. Bordetella_pertussis_strain_J019_ NZ_CP011753
304. Bordetella_pertussis_strain_J021_ NZ_CP011754
305. Bordetella_pertussis_strain_J022_ NZ_CP011244
306. Bordetella_pertussis_strain_J023_ NZ_CP011755
307. Bordetella_pertussis_strain_J024_ NZ_CP025353
308. Bordetella_pertussis_strain_J027_ NZ_CP011756
309. Bordetella_pertussis_strain_J030_ NZ_CP011757
310. Bordetella_pertussis_strain_J038_ NZ_CP012087
311. Bordetella_pertussis_strain_J039_ NZ_CP011758
312. Bordetella_pertussis_strain_J042_ NZ_CP019869
313. Bordetella_pertussis_strain_J043_ NZ_CP016887
314. Bordetella_pertussis_strain_J066_ NZ_CP026998
315. Bordetella_pertussis_strain_J068_ NZ_CP011759
316. Bordetella_pertussis_strain_J072_ NZ_CP011760
317. Bordetella_pertussis_strain_J073_ NZ_CP011761
318. Bordetella_pertussis_strain_J074_ NZ_CP017884
319. Bordetella_pertussis_strain_J076_ NZ_CP011762
320. Bordetella_pertussis_strain_J077_ NZ_CP025344
321. Bordetella_pertussis_strain_J078_ NZ_CP021401
322. Bordetella_pertussis_strain_J081_ NZ_CP017885
323. Bordetella_pertussis_strain_J085_ NZ_CP026997
324. Bordetella_pertussis_strain_J090_ NZ_CP011763
325. Bordetella_pertussis_strain_J091_ NZ_CP011764
326. Bordetella_pertussis_strain_J092_ NZ_CP026922
327. Bordetella_pertussis_strain_J093_ NZ_CP013884
328. Bordetella_pertussis_strain_J094_ NZ_CP018037
329. Bordetella_pertussis_strain_J096_ NZ_CP013951
330. Bordetella_pertussis_strain_J097_ NZ_CP013885
331. Bordetella_pertussis_strain_J098_ NZ_CP011765
332. Bordetella_pertussis_strain_J099_ NZ_CP013886
333. Bordetella_pertussis_strain_J100_ NZ_CP011766
334. Bordetella_pertussis_strain_J103_ NZ_CP013887
335. Bordetella_pertussis_strain_J104_ NZ_CP013888
336. Bordetella_pertussis_strain_J105_ NZ_CP013889
337. Bordetella_pertussis_strain_J107_ NZ_CP012088
338. Bordetella_pertussis_strain_J108_ NZ_CP017163
339. Bordetella_pertussis_strain_J109_ NZ_CP017162
340. Bordetella_pertussis_strain_J110_ NZ_CP011767
341. Bordetella_pertussis_strain_J115_ NZ_CP011768
342. Bordetella_pertussis_strain_J118_ NZ_CP026921
343. Bordetella_pertussis_strain_J120_ NZ_CP013890
344. Bordetella_pertussis_strain_J121_ NZ_CP013891
345. Bordetella_pertussis_strain_J122_ NZ_CP013892
346. Bordetella_pertussis_strain_J124_ NZ_CP013893
347. Bordetella_pertussis_strain_J129_ NZ_CP026920
348. Bordetella_pertussis_strain_J130_ NZ_CP026919
349. Bordetella_pertussis_strain_J132_ NZ_CP026918
350. Bordetella_pertussis_strain_J139_ NZ_CP025527
351. Bordetella_pertussis_strain_J148_ NZ_CP017923
352. Bordetella_pertussis_strain_J149_ NZ_CP013894
353. Bordetella_pertussis_strain_J151_ NZ_CP017161
354. Bordetella_pertussis_strain_J152_ NZ_CP013895
355. Bordetella_pertussis_strain_J153_ NZ_CP017123
356. Bordetella_pertussis_strain_J154_ NZ_CP017122
357. Bordetella_pertussis_strain_J155_ NZ_CP013896
358. Bordetella_pertussis_strain_J159_ NZ_CP013897
359. Bordetella_pertussis_strain_J160_ NZ_CP017119
360. Bordetella_pertussis_strain_J161_ NZ_CP013898
361. Bordetella_pertussis_strain_J162_ NZ_CP013899
362. Bordetella_pertussis_strain_J165_ NZ_CP017158
363. Bordetella_pertussis_strain_J169_ NZ_CP012089
364. Bordetella_pertussis_strain_J170_ NZ_CP017166
365. Bordetella_pertussis_strain_J171_ NZ_CP026992
366. Bordetella_pertussis_strain_J172_ NZ_CP017121
367. Bordetella_pertussis_strain_J173_ NZ_CP017924
368. Bordetella_pertussis_strain_J174_ NZ_CP013900
369. Bordetella_pertussis_strain_J175_ NZ_CP013901
370. Bordetella_pertussis_strain_J178_ NZ_CP013088
371. Bordetella_pertussis_strain_J179_ NZ_CP013089
372. Bordetella_pertussis_strain_J184_ NZ_CP025383
373. Bordetella_pertussis_strain_J185_ NZ_CP025343
374. Bordetella_pertussis_strain_J191_ NZ_CP013090
375. Bordetella_pertussis_strain_J192_ NZ_CP013091
376. Bordetella_pertussis_strain_J193_ NZ_CP013092
377. Bordetella_pertussis_strain_J194_ NZ_CP013093
378. Bordetella_pertussis_strain_J197_ NZ_CP013094
379. Bordetella_pertussis_strain_J198_ NZ_CP013902
380. Bordetella_pertussis_strain_J199_ NZ_CP011245
381. Bordetella_pertussis_strain_J201_ NZ_CP013095
382. Bordetella_pertussis_strain_J203_ NZ_CP017925
383. Bordetella_pertussis_strain_J204_ NZ_CP026991
384. Bordetella_pertussis_strain_J205_ NZ_CP026990
385. Bordetella_pertussis_strain_J206_ NZ_CP013903
386. Bordetella_pertussis_strain_J207_ NZ_CP018038
387. Bordetella_pertussis_strain_J208_ NZ_CP013904
388. Bordetella_pertussis_strain_J209_ NZ_CP017120
389. Bordetella_pertussis_strain_J210_ NZ_CP013905
390. Bordetella_pertussis_strain_J222_ NZ_CP017165
391. Bordetella_pertussis_strain_J223_ NZ_CP017160
392. Bordetella_pertussis_strain_J224_ NZ_CP017164
393. Bordetella_pertussis_strain_J225_ NZ_CP013096
394. Bordetella_pertussis_strain_J226_ NZ_CP017159
395. Bordetella_pertussis_strain_J230_ NZ_CP017926
396. Bordetella_pertussis_strain_J233_ NZ_CP026917
397. Bordetella_pertussis_strain_J234_ NZ_CP025348
398. Bordetella_pertussis_strain_J246_ NZ_CP026989
399. Bordetella_pertussis_strain_J247_ NZ_CP026988
400. Bordetella_pertussis_strain_J249_ NZ_CP026464
401. Bordetella_pertussis_strain_J250_ NZ_CP026463
402. Bordetella_pertussis_strain_J251_ NZ_CP026462
403. Bordetella_pertussis_strain_J252_ NZ_CP026461
404. Bordetella_pertussis_strain_J253_ NZ_CP026460
405. Bordetella_pertussis_strain_J254_ NZ_CP026459
406. Bordetella_pertussis_strain_J255_ NZ_CP026458
407. Bordetella_pertussis_strain_J256_ NZ_CP026457
408. Bordetella_pertussis_strain_J257_ NZ_CP026456
409. Bordetella_pertussis_strain_J258_ NZ_CP026455
410. Bordetella_pertussis_strain_J259_ NZ_CP026454
411. Bordetella_pertussis_strain_J260_ NZ_CP026453
412. Bordetella_pertussis_strain_J262_ NZ_CP026452
413. Bordetella_pertussis_strain_J263_ NZ_CP026451
414. Bordetella_pertussis_strain_J264_ NZ_CP026450
415. Bordetella_pertussis_strain_J266_ NZ_CP026449
416. Bordetella_pertussis_strain_J267_ NZ_CP026448
417. Bordetella_pertussis_strain_J268_ NZ_CP026447
418. Bordetella_pertussis_strain_J269_ NZ_CP026446
419. Bordetella_pertussis_strain_J270_ NZ_CP026445
420. Bordetella_pertussis_strain_J272_ NZ_CP026444
421. Bordetella_pertussis_strain_J274_ NZ_CP026443
422. Bordetella_pertussis_strain_J275_ NZ_CP026442
423. Bordetella_pertussis_strain_J276_ NZ_CP013906
424. Bordetella_pertussis_strain_J277_ NZ_CP013907
425. Bordetella_pertussis_strain_J294_ NZ_CP018039
426. Bordetella_pertussis_strain_J295_ NZ_CP017927
427. Bordetella_pertussis_strain_J296_ NZ_CP013863
428. Bordetella_pertussis_strain_J300_ NZ_CP013864
429. Bordetella_pertussis_strain_J303_ NZ_CP026987
430. Bordetella_pertussis_strain_J304_ NZ_CP018040
431. Bordetella_pertussis_strain_J305_ NZ_CP013865
432. Bordetella_pertussis_strain_J306_ NZ_CP018041
433. Bordetella_pertussis_strain_J308_ NZ_CP017167
434. Bordetella_pertussis_strain_J310_ NZ_CP017168
435. Bordetella_pertussis_strain_J311_ NZ_CP013866
436. Bordetella_pertussis_strain_J313_ NZ_CP018042
437. Bordetella_pertussis_strain_J322_ NZ_CP026441
438. Bordetella_pertussis_strain_J323_ NZ_CP026440
439. Bordetella_pertussis_strain_J330_ NZ_CP026439
440. Bordetella_pertussis_strain_J332_ NZ_CP026438
441. Bordetella_pertussis_strain_J334_ NZ_CP026437
442. Bordetella_pertussis_strain_J336_ NZ_CP026436
443. Bordetella_pertussis_strain_J354_ NZ_CP026435
444. Bordetella_pertussis_strain_J357_ NZ_CP026434
445. Bordetella_pertussis_strain_J358_ NZ_CP014212
446. Bordetella_pertussis_strain_J359_ NZ_CP026433
447. Bordetella_pertussis_strain_J360_ NZ_CP026432
448. Bordetella_pertussis_strain_J362_ NZ_CP026633
449. Bordetella_pertussis_strain_J363_ NZ_CP026431
450. Bordetella_pertussis_strain_J364_ NZ_CP026430
451. Bordetella_pertussis_strain_J365_ NZ_CP013867
452. Bordetella_pertussis_strain_J366_ NZ_CP026429
453. Bordetella_pertussis_strain_J367_ NZ_CP026472
454. Bordetella_pertussis_strain_J368_ NZ_CP026632
455. Bordetella_pertussis_strain_J369_ NZ_CP026669
456. Bordetella_pertussis_strain_J370_ NZ_CP026471
457. Bordetella_pertussis_strain_J372_ NZ_CP026668
458. Bordetella_pertussis_strain_J373_ NZ_CP026667
459. Bordetella_pertussis_strain_J374_ NZ_CP026631
460. Bordetella_pertussis_strain_J375_ NZ_CP026666
461. Bordetella_pertussis_strain_J376_ NZ_CP026470
462. Bordetella_pertussis_strain_J377_ NZ_CP014211
463. Bordetella_pertussis_strain_J378_ NZ_CP026630
464. Bordetella_pertussis_strain_J379_ NZ_CP026665
465. Bordetella_pertussis_strain_J384_ NZ_CP025073
466. Bordetella_pertussis_strain_J386_ NZ_CP026629
467. Bordetella_pertussis_strain_J390_ NZ_CP026664
468. Bordetella_pertussis_strain_J391_ NZ_CP026469
469. Bordetella_pertussis_strain_J392_ NZ_CP026468
470. Bordetella_pertussis_strain_J393_ NZ_CP026428
471. Bordetella_pertussis_strain_J401_ NZ_CP026467
472. Bordetella_pertussis_strain_J402_ NZ_CP026466
473. Bordetella_pertussis_strain_J424_ NZ_CP026628
474. Bordetella_pertussis_strain_J445_ NZ_CP017402
475. Bordetella_pertussis_strain_J446_ NZ_CP017403
476. Bordetella_pertussis_strain_J447_ NZ_CP017404
477. Bordetella_pertussis_strain_J448_ NZ_CP017405
478. Bordetella_pertussis_strain_J473_ NZ_CP021403
479. Bordetella_pertussis_strain_J522_ NZ_CP026627
480. Bordetella_pertussis_strain_J523_ NZ_CP026626
481. Bordetella_pertussis_strain_J524_ NZ_CP026625
482. Bordetella_pertussis_strain_J525_ NZ_CP026624
483. Bordetella_pertussis_strain_J526_ NZ_CP026663
484. Bordetella_pertussis_strain_J527_ NZ_CP026623
485. Bordetella_pertussis_strain_J528_ NZ_CP026622
486. Bordetella_pertussis_strain_J529_ NZ_CP026621
487. Bordetella_pertussis_strain_J546_ NZ_CP026916
488. Bordetella_pertussis_strain_J549_ NZ_CP026915
489. Bordetella_pertussis_strain_J567_ NZ_CP026914
490. Bordetella_pertussis_strain_J571_ NZ_CP026913
491. Bordetella_pertussis_strain_J582_ NZ_CP026912
492. Bordetella_pertussis_strain_J586_ NZ_CP026911
493. Bordetella_pertussis_strain_J588_ NZ_CP026910
494. Bordetella_pertussis_strain_J589_ NZ_CP026909
495. Bordetella_pertussis_strain_J603_ NZ_CP026908
496. Bordetella_pertussis_strain_J604_ NZ_CP026907
497. Bordetella_pertussis_strain_J605_ NZ_CP026906
498. Bordetella_pertussis_strain_J606_ NZ_CP026905
499. Bordetella_pertussis_strain_J614_ NZ_CP026904
500. Bordetella_pertussis_strain_J616_ NZ_CP026903
501. Bordetella_pertussis_strain_J617_ NZ_CP026902
502. Bordetella_pertussis_strain_J625_ NZ_CP022362
503. Bordetella_pertussis_strain_J628_ NZ_CP026901
504. Bordetella_pertussis_strain_J632_ NZ_CP026620
505. Bordetella_pertussis_strain_J650_ NZ_CP026619
506. Bordetella_pertussis_strain_J656_ NZ_CP026900
507. Bordetella_pertussis_strain_J662_ NZ_CP026899
508. Bordetella_pertussis_strain_J667_ NZ_CP026898
509. Bordetella_pertussis_strain_J673_ NZ_CP026618
510. Bordetella_pertussis_strain_J679_ NZ_CP026897
511. Bordetella_pertussis_strain_J680_ NZ_CP026896
512. Bordetella_pertussis_strain_J682_ NZ_CP026895
513. Bordetella_pertussis_strain_J683_ NZ_CP026894
514. Bordetella_pertussis_strain_J687_ NZ_CP026893
515. Bordetella_pertussis_strain_J690_ NZ_CP026892
516. Bordetella_pertussis_strain_J691_ NZ_CP026891
517. Bordetella_pertussis_strain_J692_ NZ_CP026890
518. Bordetella_pertussis_strain_J693_ NZ_CP026889
519. Bordetella_pertussis_strain_J695_ NZ_CP026888
520. Bordetella_pertussis_strain_J697_ NZ_CP026887
521. Bordetella_pertussis_strain_J698_ NZ_CP026886
522. Bordetella_pertussis_strain_J699_ NZ_CP026885
523. Bordetella_pertussis_strain_J700_ NZ_CP026884
524. Bordetella_pertussis_strain_J702_ NZ_CP026883
525. Bordetella_pertussis_strain_J703_ NZ_CP026882
526. Bordetella_pertussis_strain_NCTC10910_chromosome_1_ NZ_LS483398
527. Bordetella_pertussis_strain_NCTC13251_chromosome_1_ NZ_LR590467
528. Bordetella_pertussis_strain_NCTC13666_chromosome_1_ NZ_LT906471
529. Bordetella_pertussis_strain_NCTC13667_chromosome_1_ NZ_LT906484
530. Bordetella_pertussis_strain_P2013109_ NZ_CP038790
531. Bordetella_pertussis_strain_Pelita_III_ NZ_CP019957
532. Bordetella_pertussis_strain_Tohama_ NZ_CP031787
533. Bordetella_pertussis_strain_UK36_ CP031289
534. Bordetella_pertussis_strain_UK38_ CP031112
535. Bordetella_pertussis_strain_UK39_ CP031113
536. Bordetella_pertussis_strain_UK76_ CP031114
537. Bordetella_pertussis_strain_UT25Sm1_ NZ_CP015771
538. Bordetella_pertussis_strain_VA_09_ NZ_CP015765
539. Bordetella_pertussis_strain_VA_10_ NZ_CP015770
540. Bordetella_pertussis_strain_VA_145_ NZ_CP015769
541. Bordetella_pertussis_strain_VA_150_ NZ_CP015762
542. Bordetella_pertussis_strain_VA_15_ NZ_CP015766
543. Bordetella_pertussis_strain_VA_175_ NZ_CP015760
544. Bordetella_pertussis_strain_VA_18_ NZ_CP015767
545. Bordetella_pertussis_strain_VA_190_ NZ_CP015761
546. Bordetella_pertussis_strain_VA_194_ NZ_CP015759
547. Bordetella_pertussis_strain_VA_198_ NZ_CP015764
548. Bordetella_pertussis_strain_VA_52_ NZ_CP015763
549. Bordetella_pertussis_strain_VA_62_ NZ_CP015768
550. Bordetella_pertussis_strain_VS366_chromosome_I_ NZ_LS398589
551. Bordetella_pertussis_strain_VS377_chromosome_I_ NZ_LS398604
552. Bordetella_pertussis_strain_VS393_chromosome_I_ NZ_LS398590
553. Bordetella_pertussis_strain_VS401_chromosome_I_ NZ_LS398605
554. Bordetella_pertussis_strain_VS67_chromosome_I_ NZ_LS398588
